# Carbosilane Dendritic Amphiphiles from Cholesterol or Vitamin E for Micelle Formation

**DOI:** 10.3390/pharmaceutics16040451

**Published:** 2024-03-25

**Authors:** Gabriel Mencia, Sergio Algar, Tania Lozano-Cruz, Mª Ángeles Muñoz-Fernández, Elizabeth R. Gillies, Jesús Cano, Mercedes Valiente, Rafael Gómez

**Affiliations:** 1 Department of Organic and Inorganic Chemistry, Research Institute in Chemistry “Andrés M. Del Río” (IQAR), University of Alcalá, 28805 Madrid, Spain; gabi.men.ber@gmail.com (G.M.); sergioalgar@ucm.es (S.A.); tania.lozano@uah.es (T.L.-C.); jesus.cano@uah.es (J.C.); 2 Networking Research Center on Bioengineering, Biomaterials and Nanomedicine (CIBER-BBN), 28029 Madrid, Spain; mmunoz.hgugm@gmail.com; 3 Ramón y Cajal Health Research Institute (IRYCIS), 28034 Madrid, Spain; 4 Laboratory Platform (Immunology), General Universitary Hospital Gregorio Marañón (HGUGM), 28007 Madrid, Spain; 5 Spanish HIV HGM BioBank, Health Research Institute Gregorio Marañón (HGUGM), 28007 Madrid, Spain; 6 Department of Chemistry and Chemical and Biochemical Engineering, School of Biomedical Engineering, University of Western Ontario, London, ON N6G1Z1, Canada; egillie@uwo.ca; 7 Department of Analytical Chemistry, Physical Chemistry and Chemical Engineering, Research Institute in Chemistry “Andrés M. Del Río” (IQAR), University of Alcalá, 28805 Madrid, Spain

**Keywords:** carbosilane, dendron, cholesterol, tocopherol, micelle, drug encapsulation

## Abstract

Cationic dendritic amphiphiles were prepared through the linkage of interesting hydrophobic molecules such as cholesterol or vitamin E to the focal point of carbosilane dendrons. These new dendritic systems self-assembled in saline, producing micellar aggregates with hydrodynamic diameters ranging from 6.5 to 9.2 nm, and critical micelle concentrations of approximately 5 and 10 μM for second- and third-generation systems, respectively. The assemblies were able to encapsulate drugs of different charges (anionic, neutral, and cationic). Surprisingly, a 92% encapsulation efficiency for diclofenac was achieved in micelles prepared from second-generation dendrons. Toxicity measurements on peripheral blood mononuclear cells indicated different behavior depending on the generation, corresponding to the micellar regime. In contrast to the third-generation system, the second-generation system was non-toxic up to 20 μM, opening a window for its use in a micellar regimen, thereby operating as a drug delivery system for different biomedical applications.

## 1. Introduction

Classical surfactants are substances formed using a hydrocarbon chain attached to an ionic polar group. Of these, the oldest used are fatty acids which are biomolecules that make up the main components of fats and consist of a carboxylic acid group attached to a hydrocarbon chain that can be of different lengths and degrees of saturation. However, other naturally occurring compounds are able to form amphiphilic systems, and, therefore, its aggregates are cholesterol and vitamin E.

Cholesterol is an essential steroid lipid in the cytoplasmic membrane, where it fulfills a very important structural function, giving rise to the final physical–chemical properties of the membrane. In addition, it is involved in biosynthetic hormone processes, such as those of testosterone and progesterone, as well as vitamin D. In the field of micellar aggregates, Haberland and Reynolds demonstrated in the 1970s that cholesterol was an amphiphilic molecule capable of forming micelles at concentrations in the order of nanomolars [1]. Therefore, in recent years, many amphiphilic structures derived from cholesterol have emerged by modifying its polar zone to improve its solubility and offer new features depending on the desired application (gene therapy, drug encapsulation, antitumor therapy, etc.) [2,3,4]. In addition, in the last decade, so-called quatsomes have been developed, where cholesterol plays a fundamental role in helping other surfactants to form more complex systems [5]. In particular, cholesterol interacts with the hydrocarbon tail of the surfactant cetyltrimethylammonium bromide (CTAB) through hydrophobic interactions. This interaction causes the non-covalent association of the two molecules to behave as a single unit, resulting in geometry changes from conical to cylindrical and favoring the formation of vesicles. Therefore, the addition of different proportions of cholesterol causes a change from classical spherical micelles of CTAB to vesicles.

Although the number of amphiphilic systems that contain cholesterol is extensive, there are only a few examples of its use to generate classic amphiphilic head/tail dendritic systems. For instance, bis-MPA dendrons were reported, which were capable of micelle formation at *c.a.* 5 μM with diameters of approximately 7 and 15 nm for a second-generation system, depending on whether they had one or two cholesterol groups at the focal point (tail), respectively [6,7,8]. Another example involved poly(amidoamine) (PAMAM)-type dendrons, with a critical micellar concentration (CMC) of 20 µM, and micelle diameters of about 115–130 nm [9,10]. In both cases, the dendritic skeletons had relatively hydrophilic characteristics, and the studies were focused on the delivery of nucleic acids with cationic micelles. In both cases, interaction with DNA occurred, which allowed these systems to be used as gene therapy carriers.

Vitamin E is a generic term that includes a total of eight biologically active compounds with similar functions. All of these compounds contain a chromanol ring and differ in their degree of methylation of the aromatic ring and in the saturation of the side chain, giving rise to the derivatives α-, β-, γ- and Δ-tocopherol and -tocotrienol. These molecules, encompassed under the name vitamin E, are the most abundant liposoluble antioxidants in cells, and their main function is to break the chain of reactions that gives rise to the oxidation of lipids [11,12]. Among them, α-tocopherol has the greatest bioavailability and activity. Numerous scientific studies in which d-α-tocopherol was used in the synthesis of amphiphilic molecules, as a lipophilic component for the generation of micelles, have been reported [13,14,15,16]. These amphiphilic systems almost always presented poly(ethylene glycol) (PEG) as the hydrophilic unit [17,18,19,20]. Another example was that formed with inulin polysaccharide as a hydrophilic part [21]. These systems with CMCs between 3 and 75 µM were able to successfully encapsulate curcumin and celecoxib with efficiencies of 37 and 52%, respectively.

Regarding its use in dendritic systems, d-α-tocopherol covalently linked to the periphery of PAMAM dendrons was reported for different biomedical applications [22,23,24]. In the specific field of dendritic micelles, d-α-tocopherol as a hydrophobic component, was bound to the periphery of poly-lysine type hydrophilic dendrons with PEG at the focal point [25,26]. These dendrons had CMCs between 1.3 and 1.5 µM, and their micelles diameters ranged from 10 to 20 nm. In addition, these micelles were able to encapsulate proteins for biomolecular transport applications. However, no amphiphilic dendrons have been reported in the literature with the classical head/tail structure where the periphery of the dendron served as the hydrophilic head group, and the tail composed of a d-α-tocopherol molecule was attached to the focal point.

In general terms, dendritic micelles have been shown to exhibit advantages compared to those composed of conventional surfactants. For instance, they can exhibit better stability in solution and lower CMCs, in addition to their multivalency that allows enhanced interactions with biological systems compared to individual small molecules [27,28,29,30]. In this sense, we have recently reported the design of new amphiphilic carbosilane dendrons with pH-dependent behavior based on the presence of carboxylate groups at the periphery and a fatty acid in the focal point to encapsulate hydrophilic and hydrophobic drugs [31]. Here, we present our results concerning the formation of carbosilane dendritic micelles using cholesterol or vitamin E (d-α-tocopherol) as the hydrophobic tail at the focal point of the dendritic scaffold and hydrophilic ammonium groups at the periphery, along with their viability for drug encapsulation.

## 2. Materials and Methods

### 2.1. Materials

**Figure 1:**
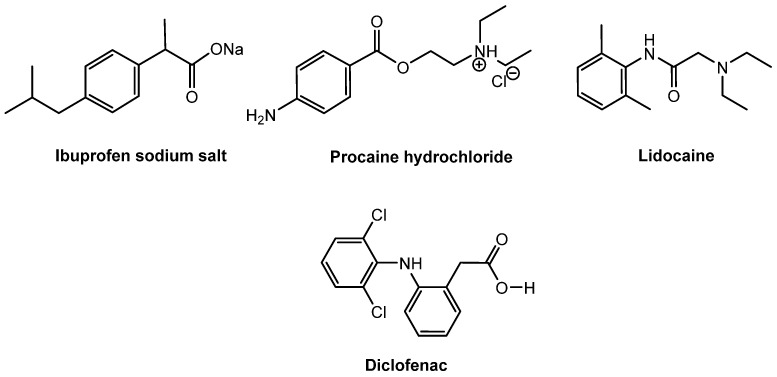
Molecular structures of selected drugs.

Reagents were purchased from commercial suppliers and used without further purification. Solvents were dried and distilled under an argon prior to use, unless specified otherwise. The dendron precursors NH2Gn(SNMe2)m (n = 1, m = 2 (i); n = 2, m = 4 (ii); n = 3, m = 8 (iii)), BrGn(V)m (n = 1, m = 2 (iv); n = 2, m = 4 (v); n = 3, m = 8 (vi)) [32] were synthesized as previously reported. *Click* reactions (thiol-ene addition) were carried out employing a HPK 125 W mercury lamp (Heraeus Nobleligth; λ_max_ = 365 nm). Hydrophilic drugs including ibuprofen, procaine and lidocaine (the latter in its neutral form), as well as diclofenac as a hydrophobic drug, were purchased from commercial sources (Figure 1).

### 2.2. Analytical and Spectroscopic Techniques

**NMR spectroscopy.** NMR spectra were recorded with a Varian Unity-300, Varian Mercury-300 or Bruker 400 Ultrashield spectrometer. All chemical shifts were determined using the residual solvent signal.

**Mass spectrometry.** The measurements were conducted employing an Agilent 6210 TOF LC-ESI-MS system. Methanol (1% formic acid) served as eluent solvent for the positive mode while methanol/water was utilized for the negative mode.

**Elemental analysis.** Elemental analyses of C, H, and S were obtained on a LECO CHNS-932 microanalyzer.

**Surface tension measurements.** The measurements were carried using the ring method in a Lauda TE 2/3 tensiometer. The optimized NaI concentration was determined in aqueous solutions containing 1 mM of dendron with a salt concentration ranging from 0 to 30 mM. The critical micellar concentration (CMC) was determined using aqueous solutions of dendrons ranging from 1 to 100 µM with NaI at 40 mM.

**Dynamic light scattering.** The assembly diameters were determined utilizing a Nano ZS zetasizer from Malvern Instruments Ltd, Malvern, United Kingdom. All measurements were carried out at 25 °C in non-reusable plastic cells. The data are presented as the mean ± standard deviation of three separate experiments.

**UV-Vis spectroscopy.** Spectroscopic measurements were performed in a UVIKON 941 Plus spectrophotometer at 25 °C with quartz cells.

**High-performance liquid chromatography (HPLC)**. HPLC analyses were performed with an Agilent 1200 HPLC with DAD detector (Model G1315D).

### 2.3. Protocols for Drug Encapsulation

**Hydrophilic drug encapsulation.** Encapsulation was determined quantitatively via absorbance measurements of saline aqueous solutions (NaI 40 mM) at 1.5 mM of ibuprofen sodium salt, 1.5 mM of lidocaine, and 10 µM of procaine hydrochloride in the presence of dendrons in the range of 0.5–50 µM.

**Hydrophobic drug encapsulation.** Drug encapsulation efficiency was determined by analyzing samples of the micellar formulations as follows. Briefly, **5** or **17** (0.5–5.5 mM) and diclofenac (1 mM) were co-dissolved in 100 μL of methanol. After 4 h of stirring, 2 mL of aqueous saline solution (NaI 40 mM) was added. The mixture was stirred for 24 h at room temperature to allow for the evaporation of the organic solvent. The suspension was then filtered (0.2 μm CA filter), and the filter was washed with methanol (3 mL) to determinate non-encapsulated diclofenac. Both the micellar solution and the non-encapsulated diclofenac were analyzed using HPLC. The drug encapsulation efficiency was calculated using the following equation:

*Encapsulation Efficiency* (*EE*,%) = *Weight of loaded drug/Weight of drug initially added* × 100

**In vitro release profile**. In vitro release of diclofenac from the diclofenac-loaded micelles (**5** + diclofenac) was studied using the dialysis bag method. Briefly, 1 mL of drug-micelle solution in phosphate-buffered saline (PBS, 10 mM, pH = 7.4) was placed in a regenerated cellulose dialysis bag (MWCO: 1 kDa), which was incubated in 250 mL of PBS at 37 °C. At predetermined time points, 100 μL of the dialysis bag was taken with buffer replacement to maintain the constant volume. The samples were analyzed using HPLC. A control experiment to determine the release of the free drug in 1 mL of acetonitrile-water (1:3) was also carried out as described above.

### 2.4. Biological Experiments

**Viability studies.** MTT assay was performed in the selected cell line to evaluate the biocompatibility of the dendrons. The assays were carried out on peripheral blood mononuclear cells (PBMCs), which were treated at concentrations ranging from 0.5 to 50 µM of the cationic dendron families and grown in PRMI medium supplemented with 10% of FBS. The cells were plated in a p96 multiwell plate and exposed to dendrons at concentrations ranging from 0.5 to 50 µM for 48 hours. Cells were treated after incubation with 0.5 mg/mL of MTT for 2 h, and crystals were dissolved in DMSO. Absorbance was measured at 570/690 nm, and viability was expressed relative to untreated cells. DMSO was used as a death control and untreated cells as the cell viability control. Data are presented as mean ± SD of three single experiments performed in triplicate**.** GraphPad software Prism v.5.0 (GraphPad Software, San Diego, CA, USA) was used for the statistical analysis of the results. Data with three replicates are displayed as bars ±SD. A *p*-value of ≤ 0.05 was considered to be statistically significant (* *p* < 0.05; ** *p* < 0.01; *** *p* < 0.001).

### 2.5. Synthesis and Characterization

A selection of the synthetic procedures based on first-generation dendrons are reported in this section. For the synthesis of the second and third generations and other compounds, see the Supporting Information.

**Synthesis of ChG_1_(NMe_2_)_2_ (1).**

An aqueous solution (3 mL) of cholesteryl chloroformate (0.772 g; 1.72 mmol) was added dropwise to a methanolic solution (2 mL) of dendron NH_2_G_1_(SNMe_2_)_2_ (**i**) (1.5 g; 1.72 mmol) [32] and NEt_3_ (0.28 mL; 2.0 mmol) under vigorous stirring. A total of 30 min later, volatiles were removed, and CH_2_Cl_2_/H_2_O extraction was carried out. The organic phase was evaporated and the crude product was purified using size-exclusion chromatography in acetone, yielding the desired dendron **1** as a yellowish oil (53%).

^1^H-NMR (CDCl_3_): δ (ppm) -0.03 (s, 3H, -Si(C*H_3_*)-), 0.49–0.55 (m, 6H, -NCH_2_CH_2_CH_2_C*H_2_*Si-, -SiC*H_2_*CH_2_S-), 0.62 (s, 3H, C*H_3_*C-, cholesterol), 0.81 (d, 6H, C*H_3_*CH-, cholesterol), 0.86 (d, 3H, C*H_3_*CH-, cholesterol), 0.95 (s, 3H, C*H_3_*C-, cholesterol), 0.84–2.00 (m, 32H, -C*H_2_*-, -C*H*-, cholesterol, -NCH_2_C*H_2_*C*H_2_*CH_2_Si-), 2.20 (m, 12H, -N(C*H_3_*)_2_), 2.50 (m, 12H, -SiCH_2_C*H_2_*S-, -SC*H_2_*CH_2_N-, -SCH_2_C*H_2_*N-), 3.10 (m, 2H, -NC*H_2_*CH_2_CH_2_CH_2_Si-), 4.42 (m, 1H, -C*H*CONH-), 4.81 (m, 1H, -CON*H*-), 5.30 (m, 1H, -C=C*H*CH_2_-, cholesterol). ^13^C-NMR (CDCl_3_): δ (ppm) -5.32 (-Si(*C*H_3_)-), 13.3 (-NCH_2_CH_2_CH_2_*C*H_2_Si-), 14.5 (-Si*C*H_2_CH_2_S-), 11.8, 18.7, 19.3, 21.0, 22.5, 22.8, 23,8, 24.2, 27.9, 28.2, 31.8, 35.7, 36.1, 36.5, 36.9, 38,5, 39.4, 39.7, 42.2, 49.9, 56.0, 56.6 (CH_3_-, -CH_2_-, -CH-, -C-, cholesterol), 20.9 (-NCH_2_CH_2_*C*H_2_CH_2_Si-), 27.6 (-SiCH_2_*C*H_2_S-), 29.7 (-S*C*H_2_CH_2_N-), 33.8 (-NCH_2_*C*H_2_CH_2_CH_2_Si-), 40.4 (-N*C*H_2_CH_2_CH_2_CH_2_Si-), 45.3 (-N(*C*H_3_)_2_), 59.2 (-SCH_2_*C*H_2_N-), 74.0 (-*C*HCONH-), 122.2 (-C=*C*HCH_2_-, cholesterol), 139.6 (-*C*=CHCH_2_-, cholesterol), 155.9 (-*C*ONH-). Mass spectrometry: [M+H]^+^ = 792.5918 Da (calcd. = 792.5925 Da.). Elemental analysis C_45_H_85_N_3_O_2_S_2_Si (792.40 g/mol): calcd. = C, 68.21; H, 10.81; N, 5.30; O, 4.04; S, 8.09; Si, 3.54. Found = C, 67.79; H, 10.62; N, 4.41; S, 8.21.

**Synthesis of ChG1(NMe3I)2 (4).**

Iodomethane (0.10 mL, 1.61 mmol) was added dropwise to a solution of dendron ChG_1_(NMe_2_)_2_ (**1**) (0.50 g, 0.63 mmol) in 100 mL of THF. The reaction was stirred at room temperature for 4 h, and then the solvent was evaporated, and the product was washed with Et_2_O (3x10 mL). The desired dendron was obtained as a yellow solid (94%).

^1^H-NMR (CD_3_OD): δ (ppm) 0.15 (s, 3H, -Si(C*H_3_*)-), 0.62–0.74 (m, 6H, -NCH_2_CH_2_CH_2_C*H_2_*Si-, -SiC*H_2_*CH_2_S-), 0.76 (s, 3H, C*H_3_*C- cholesterol), 0.92 (d, 6H, C*H_3_*CH-, cholesterol), 0.98 (d, 3H, C*H_3_*CH-, cholesterol), 1.08 (s, 3H, C*H_3_*C-, cholesterol), 0.95–2.43 (m, 32H, -C*H_2_*-, -C*H*-, cholesterol, -NCH_2_C*H_2_*C*H_2_*CH_2_Si-), 2.78 (m, 4H, -SiCH_2_C*H_2_*S-), 3.02 (m, 4H, -SC*H_2_*CH_2_N-), 3.13 (m, 2H, -NC*H_2_*CH_2_CH_2_CH_2_Si-), 3.23 (s, 18H, -N(C*H_3_*)_3_), 3.66 (m, 4H, -SCH_2_C*H_2_*N-), 4.40 (m, 1H, -C*H*CONH-), 5.42 (m, 1H, -C=C*H*CH_2_-, cholesterol). ^13^C-RMN (CD_3_OD): δ (ppm) -4.90 (-Si(*C*H_3_)-), 14.1 (-NCH_2_CH_2_CH_2_*C*H_2_Si-), 15.6 (-Si*C*H_2_CH_2_S-), 12.4, 19.3, 22.3, 23.0, 23.2, 25.0, 25.4, 28.9, 29.1, 29.3, 33.0, 33.2, 37.1, 37.4, 37.8, 38.3, 39.8, 40.7, 43.5, 51.6, 57.5, 58.1 (CH_3_-, -CH_2_-, -CH-, -C-, cholesterol), 22.0 (-NCH_2_CH_2_*C*H_2_CH_2_Si-), 32.3 (-SiCH_2_*C*H_2_S-), 34.7 (-NCH_2_*C*H_2_CH_2_CH_2_Si-), 38.5 (-S*C*H_2_CH_2_N-), 41.1 (-N*C*H_2_CH_2_CH_2_CH_2_Si-), 53.9 (-N(*C*H_3_)_3_), 67.1 (-SCH_2_*C*H_2_N-), 75.4 (-*C*HCONH-), 122.4 (-C=*C*HCH_2_-, cholesterol), 141.3 (-*C*=CHCH_2_-, cholesterol), 158.7 (-*C*ONH-). Mass spectrometry: [M-I]^+^ = 948.5323 Da (calcd. = 948.5367 Da). Elemental analysis C_47_H_91_I_2_N_3_O_2_S_2_Si (1076.28 g/mol): calcd. = C, 52.45; H, 8.52; I, 23.58; N, 3.90; O, 2.97; S, 5.96; Si, 2.61. Found. = C, 52.11; H, 7.79; N, 3.52; S, 5.54.

**Synthesis of EG_1_(V)_2_ (7).**

A mixture of BrG_1_(V)_2_ (**iv**) [32] (1.0 g; 4.29 mmol), d-α-tocopherol (1.85 g; 4.29 mmol) in 50 mL of acetone and then K_2_CO_3_ (1.20 g; 8.68 mmol) and 18-crown-6 (0.092 g; 0.35 mmol) were added and stirred at 90 °C for 48 h. Then, the solvent was removed under a vacuum, and the purification was carried out through H_2_O/Et_2_O extraction. The organic fraction was dried with MgSO_4_ before the solvent was removed. Dendron **7** was obtained as an orange oil (87%). 

^1^H-NMR (CDCl_3_): δ (ppm) 0.18 (s, 3H, -Si(C*H_3_*)-), 0.75 (m, 2H, -OCH_2_CH_2_CH_2_C*H_2_*Si-), 0.88 (m, 12H, C*H_3_*CH-), 1.00–1.68 (m, 23H, -C*H_2_*-, -C*H*-, tocopherol, -OCH_2_CH_2_C*H_2_*CH_2_Si-), 1.25 (s, 3H, -CH_2_C(C*H_3_*)OC_Ar_-), 1.83 (m, 4H, -C*H_2_*C(CH_3_)OC_Ar_-, -OCH_2_C*H_2_*CH_2_CH_2_Si-), 2.10 (s, 3H, C*H_3_*C_Ar_-), 2.13 (s, 3H, C*H_3_*C_Ar_-), 2.17 (s, 3H, C*H_3_*C_Ar_-), 2.58 (t, 2H, -C_Ar_C*H_2_*CH_2_-), 3.64 (t, 2H, -OC*H_2_*CH_2_CH_2_CH_2_Si-), 5.73 (m, 2H, -SiC*H*=CH_2_), 6.08 (m, 4H, -SiCH=C*H_2_*). ^13^C-NMR (CDCl_3_): δ (ppm) -5.22 (-Si(*C*H_3_)-), 11.9, 12.0, 12.8 (*C*H_3_C_Ar_-), 14.1 (-OCH_2_CH_2_CH_2_*C*H_2_Si-), 19.7, 19.8, 20.5, 21.1, 22.7, 22.8, 23.9, 24.5, 24.9, 28.0, 31.4, 32.7, 32.8, 37.3, 37.5, 39.4, 40.1 (-*C*H_3_-, -*C*H_2_-, -*C*H-, tocopherol), 30.0 (-OCH_2_CH_2_*C*H_2_CH_2_Si-), 34.0 (-OCH_2_*C*H_2_CH_2_CH_2_Si-), 72.6 (-O*C*H_2_CH_2_CH_2_CH_2_Si-), 74.6 (-CH_2_(CH_3_)*C*OC_Ar_-), 117.3 (-*C_Ar_*CH_2_CH_2_-), 122.6, 125.6, 127.6 (CH_3_*C_Ar_*-), 132.7 (-SiCH=*C*H_2_), 136.7 (-Si*C*H=CH_2_), 147.4 (-*C_Ar_*OC-), 148.2 ((-*C_Ar_*OCH_2_-). Mass spectrometry: [M+H]^+^ = 583.4937 Da (calcd. = 583.4905 Da). Elemental analysisC_38_H_66_O_2_Si (583.03 g/mol): calcd. = C, 78.28; H, 11.41; O, 5.49; Si, 4.82. Found = C, 77.92; H, 11.05.

**Synthesis of EG_1_(SNMe_2_·HCl)_2_ (10).**

Following a similar procedure described for the starting material **i** [32], dendron **10** was obtained as a white solid (95%) using EG_1_(V)_2_ (**7**) (1.0 g; 1.72 mmol), SH(CH_2_)_2_SNMe_2_·HCl (0.511 g; 3.61 mmol), DMPA (0.052 g; 0.20 mmol).

^1^H-NMR (CD_3_OD): δ (ppm) 0.14 (s, 3H, -Si(C*H_3_*)-), 0.64–0.80 (m, 6H, -OCH_2_CH_2_CH_2_C*H_2_*Si-, -SiC*H_2_*CH_2_S-), 0.88 (m, 12H, C*H_3_*CH-), 0.95–1.65 (m, 23H, -C*H_2_*-, -C*H*-, tocopherol, -OCH_2_CH_2_C*H_2_*CH_2_Si-), 1.21 (s, 3H, -CH_2_C(C*H_3_*)OC_Ar_-), 1.79 (m, 4H, -C*H_2_*C(CH_3_)OC_Ar_-, -OCH_2_C*H_2_*CH_2_CH_2_Si-), 1.94–2.19 (m, 9H, C*H_3_*C_Ar_-), 2.56 (t, 2H, -C_Ar_C*H_2_*CH_2_-), 2.75 (m, 4H, -SiCH_2_C*H_2_*S-), 2.94 (m, 12H, -N(C*H_3_*)_2_HCl), 2.99 (m, 4H, -SC*H_2_*CH_2_N-), 3.39 (m, 4H, -SCH_2_C*H_2_*N-), 3.62 (t, 2H, -OC*H_2_*CH_2_CH_2_CH_2_Si-).

**Synthesis of EG_1_(SNMe_2_)_2_ (13).**

Following the procedure described for **1**, dendron **13** was obtained as a yellow oil (49%) using EG_1_(SNMe_2·_HCl)_2_ (**10**) (1.5 g; 1.72 mmol) and Na_2_CO_3_ (0.532 g; 5.02 mmol).

^1^H-NMR (CDCl_3_): δ (ppm) 0.03 (s, 3H, -Si(C*H_3_*)-), 0.63 (m, 2H, -OCH_2_CH_2_CH_2_C*H_2_*Si-), 0.88 (m, 12H, C*H_3_*CH-), 0.92 (m, 4H, -SiC*H_2_*CH_2_S-), 0.97–1.62 (m, 23H, -C*H_2_*-, -C*H*-, tocopherol, -OCH_2_CH_2_C*H_2_*CH_2_Si-), 1.20 (s, 3H, -CH_2_C(C*H_3_*)OC_Ar_-), 1.77 (m, 4H, -C*H_2_*C(CH_3_)OC_Ar_-, -OCH_2_C*H_2_*CH_2_CH_2_Si-), 2.05 (s, 3H, C*H_3_*C_Ar_-), 2.09 (s, 3H, C*H_3_*C_Ar_-), 2,.13 (s, 3H, C*H_3_*C_Ar_-), 2.22 (m, 12H, -N(C*H_3_*)_2_), 2.41–2.68 (m, 14H, -C_Ar_C*H_2_*CH_2_-, -SiCH_2_C*H_2_*S-, -SC*H_2_*CH_2_N-, -SCH_2_C*H_2_*N-), 3.60 (t, 2H, -OC*H_2_*CH_2_CH_2_CH_2_Si-). ^13^C-NMR (CDCl_3_): δ (ppm) -5.55 (-Si(*C*H_3_)-), 11.6, 11.7, 12.6 (*C*H_3_C_Ar_-), 14.2–14.5 (-Si*C*H_2_-), 19.5, 19.6, 20.4, 20.8, 22.5, 22.6, 23.7, 24.2, 24.6, 27.8, 31.1, 32.5, 32.6, 37.1, 37.3, 39.2, 39.8 (-*C*H_3_-, -*C*H_2_-, -*C*H-, tocopherol), 27.5 (-SiCH_2_*C*H_2_S-), 29.6 (-S*C*H_2_CH_2_N-), 29.8 (-OCH_2_CH_2_*C*H_2_CH_2_Si-), 33.9 (-OCH_2_*C*H_2_CH_2_CH_2_Si-), 49.2 (-N(*C*H_3_)_2_), 59.1 (-SCH_2_*C*H_2_N-), 72.2 (-O*C*H_2_CH_2_CH_2_CH_2_Si-), 74.5 (-CH_2_(CH_3_)*C*OC_Ar_-), 117.2 (-*C_Ar_*CH_2_CH_2_-), 122.5, 125.5, 127.5 (CH_3_*C_Ar_*-), 147.4 (-*C_Ar_*OC-), 148.1 (-*C_Ar_*OCH_2_-). Mass spectrometry: [M+H]^+^ = 793.6105 Da (calcd. = 793.6129 Da). Elemental analysis C_46_H_88_N_2_O_2_S_2_Si (793.43 g/mol): calcd. = C, 69.64; H, 11.18; N, 3.53; O, 4.03; S, 8.08; Si, 3.54. Found. = C, 69.84; H, 10.73; N, 3.04; S, 7.61.

**Synthesis** **of EG_1_(NMe_3_I)_2_ (16).**

Following the procedure described for **4**, dendron **16** was obtained as a yellow solid (96%) using EG_1_(NMe_2_)_2_ (**13**) (0.50 g, 0.63 mmol) and MeI (0.10 mL, 1.61 mmol).

^1^H-NMR (CD_3_OD): δ (ppm) 0.18 (s, 3H, -Si(C*H_3_*)-), 0.81 (m, 2H, -OCH_2_CH_2_CH_2_C*H_2_*Si-), 0.91 (m, 12H, C*H_3_*CH-), 1.06 (m, 4H, -SiC*H_2_*CH_2_S-), 1.09–1.74 (m, 23H, -C*H_2_*-, -C*H*-, tocopherol, -OCH_2_CH_2_C*H_2_*CH_2_Si-), 1.27 (s, 3H, -CH_2_C(C*H_3_*)OC_Ar_-), 1.84 (m, 4H, -C*H_2_*C(CH_3_)OC_Ar_-, -OCH_2_C*H_2_*CH_2_CH_2_Si-), 2.09 (m, 3H, C*H_3_*C_Ar_-), 2.16 (m, 3H, C*H_3_*C_Ar_-), 2.19 (m, 3H, C*H_3_*C_Ar_-), 2.63 (t, 2H, -C_Ar_C*H_2_*CH_2_-), 2.81 (m, 4H, -SiCH_2_C*H_2_*S-), 3.02 (m, 4H, -SC*H_2_*CH_2_N-), 3.21 (m, 18H, -N(C*H_3_*)_3_), 3.68 (m, 6H, -OC*H_2_*CH_2_CH_2_CH_2_Si-, -SCH_2_C*H_2_*N-). ^13^C-NMR (CD_3_OD): δ (ppm) -4,75 (-Si(*C*H_3_)-), 12.1, 12.3, 13.2 (*C*H_3_C_Ar_-), 14.5 (-OCH_2_CH_2_CH_2_*C*H_2_Si-), 15.7 (-Si*C*H_2_CH_2_S-), 20.3, 21.6, 21.7, 21.9, 23.1, 23.2, 23.3, 24.3, 25.4, 28.9, 32.7, 33.7, 33.9, 37.0, 38.3, 38.4, 38.5, 40.5 (-*C*H_3_-, -*C*H_2_-, -*C*H-, tocopherol), 25.9 (-SiCH_2_*C*H_2_S-), 29.1 (-S*C*H_2_CH_2_N-), 31.7 (-OCH_2_CH_2_*C*H_2_CH_2_Si-), 35.2 (-OCH_2_*C*H_2_CH_2_CH_2_Si-), 53.9 (-N(*C*H_3_)_3_), 67.2 (-SCH_2_*C*H_2_N-), 73.6 (-O*C*H_2_CH_2_CH_2_CH_2_Si-), 75.8 (-CH_2_(CH_3_)*C*OC_Ar_-), 118.9 (-*C_Ar_*CH_2_CH_2_-), 123.7, 126.8, 127.9 (CH_3_*C_Ar_*-), 148.9 (-*C_Ar_*OC-), 149.5 (-*C_Ar_*OCH_2_-). Mass spectrometry: [M-I]^+^ = 949.5547 Da (calcd. = 949.5571 Da). Elemental analysistal C_48_H_94_I_2_N_2_O_2_S_2_Si (1077.31 g/mol): calcd. = C, 53.52; H, 8.80; I, 23.56; N, 2.60; O, 2.97; S, 5.95; Si, 2.61. Found= C, 53.14; H, 8.27; N, 2.20; S, 5.46.3. 

## 3. Results and Discussion

### 3.1. Synthesis of Amphiphilic Dendrons

Amphiphilic dendrons with different functional groups at their peripheries and focal points were prepared using protocols developed in our research group for analogous dendritic systems [32]. Hydrophobic molecules with self-assembly inducing capabilities, including cholesterol or d-α-tocopherol, were incorporated at the focal points. Then, nucleophilic acyl substitution reactions were used to functionalize the dendrimer peripheries with hydrophilic trimethylammonium groups which would exhibit cationic charge regardless of the pH. 

#### 3.1.1. Cationic Dendrons with Cholesterol at the Focal Point

**Scheme 1:**
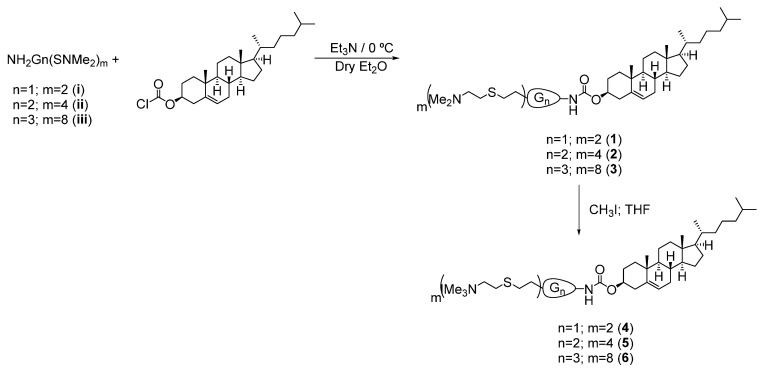
Synthesis of ammonium-terminated dendrons with cholesterol at the focal point.

The conjugation of the cholesterol molecule at the focal point using a carbamate linkage was performed by employing a chloroformate derivative of cholesterol (Scheme 1). The reactions with the chloroformate were carried out using primary amine-containing dendrons NH_2_G_n_(SNMe_2_)_m_ (n = 1, m = 2 (**i**); n = 2, m = 4 (**ii**); n = 3, m = 8 (**iii**)), previously prepared in good yields, following Gabriel and thiol-ene reactions [32]. After a typical extraction workup, the desired cholesterol-modified dendrons ChG_n_(NMe_2_)_m_ (n = 1, m = 2 (**1**); n = 2, m = 4 (**2**); n = 3, m = 8 (**3**)) were obtained as colorless oils with good yields (70–80%). The reactions were monitored using ^1^H-NMR spectroscopy (Figure S1) where signals at 3.13 ppm corresponding to the methylene group attached to the nitrogen atom, confirmed bond formation. In addition, resonances attributed to the cholesterol fragment were observed at 5.34 ppm (-C=CH-) and 4.47 ppm (-CH(O)-). The peak corresponding to the proton in the carbamate N-H appeared at 4.59 ppm but exhibited slight concentration-dependent shifting. In the ^13^C-NMR spectrum (Figure S2), the resonances ascribed to the same units were observed at 40.6 ppm (-CH_2_N-), 74.1 ppm (-CH(O)-), and 139.7 and 122.3 ppm (-C=CH-). In addition, the characteristic signal of the carbonyl group was seen at 155.9 ppm. 

**Figure 2:**
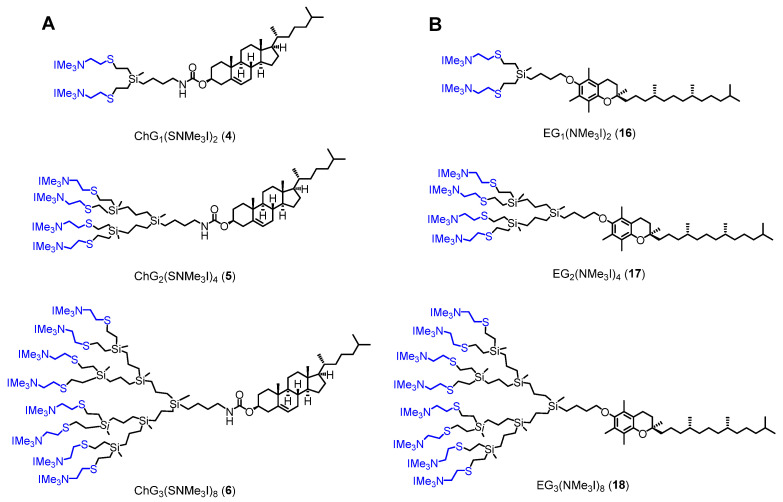
Amphiphilic ammonium-terminated dendrons with cholesterol (**4**–**6**; (**A**)) or d-α-tocopherol (**16**–**18**; (**B**)) at the focal point.

Finally, the preparation of ammonium-terminated dendrons was achieved through the reaction of **1**–**3** with an excess of MeI in dry THF (Scheme 1). Amphiphilic dendrons with cholesterol at the focal point ChG_n_(NMe_3_I)_m_ (n = 1, m = 2 (**4**); n = 2, m = 4 (**5**); n = 3, m = 8 (**6**)) were obtained as yellow solids with excellent yield (>90%). ^1^H-NMR spectroscopic evaluation supported the correct formation of the desired compounds based on the visualization of the deshielding effect observed for moieties adjacent to the nitrogen atom (Figure S3). After the reaction, the resonances appeared at 2.78, 3.02, and 3.66 ppm for methylene groups in -SiCH_2_C*H*_2_S-, -SC*H*_2_CH_2_N-, and -SCH_2_C*H*_2_N-, respectively. In addition, the methyl unit was observed at 3.23 ppm as a singlet. In the ^13^C-NMR spectrum (Figure S4), the same methylene fragments were observed at 32.3 (-SiCH_2_*C*H_2_S-), 38.5 (-S*C*H_2_CH_2_N-), and 67.1 (-SCH_2_*C*H_2_N-) ppm, while the carbon atom in the methyl moiety (-NMe_3_I) was deshielded and appeared at 53.9 ppm. Figure 2A shows the full structures of amphiphilic cationic compounds **4**–**6**.

#### 3.1.2. Cationic Dendrons with d-α-Tocopherol at the Periphery

**Scheme 2:**
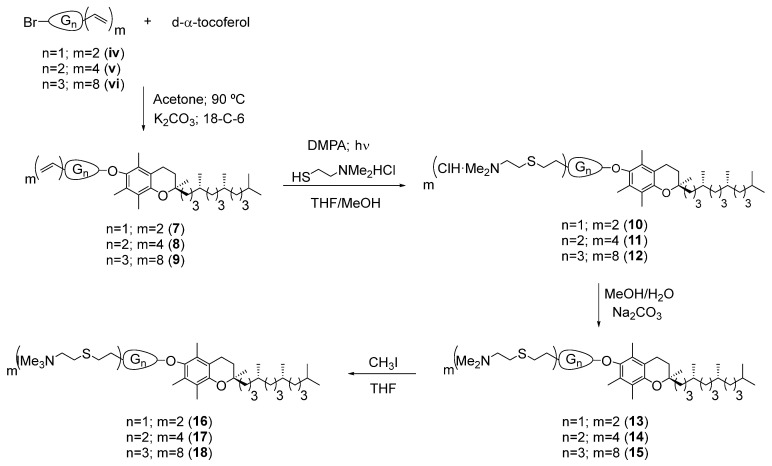
Synthesis of ammonium-terminated dendrons with d-α-tocopherol at the focal point.

The d-α-tocopherol, also called vitamin E, was incorporated at the dendron focal points through ether linkages. First, dendrons with a bromine atom at the focal point BrG_n_(V)_m_ (n = 1, m = 2 (**iv**); n = 2, m = 4 (**v**); n = 3, m = 8 (**vi**)) were selected as precursors [32]. Then, following a previously reported protocol, a solution of dendrons **iv**-**vi**, d-α-tocopherol, K_2_CO_3,_ and crown ether (18-crown-6) was stirred at 90 °C (Scheme 2) [32]. The desired compounds EG_n_(V)_m_ (n = 1, m = 2 (**7**); n = 2, m = 4 (**8**); n = 3, m = 8 (**9**)) were obtained as viscous oils with very good yields (85–95%). In the ^1^H-NMR spectra, the disappearance of the resonance corresponding to the hydroxyl group in free d-α-tocopherol and the shifting of the peak corresponding to the adjacent methylene group up to 3.64 ppm confirmed the conjugation (Figure S5). In addition, the chemical shifts of the peaks corresponding to the methyl groups on the aromatic ring of d-α-tocopherol were affected by the ether bond formation, appearing at 2.09, 2.13, and 2.17 ppm. In the ^13^C-NMR spectra (Figure S6), the most characteristic signals corresponded to the methylene group in the dendron (72.6 ppm) and the aromatic carbon atom (148.2 ppm) of d-α-tocopherol, both of which were directly bonded to the oxygen atom of the ether, and were consequently deshielded.

The next step was the functionalization of the peripheral vinyl groups of the dendrons via click chemistry (Scheme 2). A methanolic solution of thiol derivative 2-(dimethylamino)ethanethiol hydrochloride (SH(CH_2_)_2_NMe_2_·HCl) and DMPA photoinitiator was added in two portions to a solution of compounds **7**–**9** in THF. Irradiation with UV light after each addition for 2 h resulted in the cationic compounds EG_n_(NMe_2_·HCl)_m_ (n = 1, m = 2 (**10**); n = 2, m = 4 (**11**); n = 3, m = 8 (**12**)) as orange solids soluble in MeOH without further purification. The reaction was monitored by loss of alkenyl resonances. The new signals corresponding to -SiCH_2_C*H*_2_S-, -SC*H*_2_CH_2_N-, and -C*H*_2_NMe_2_·HCl were observed around 2.75, 2.99, and 3.39 ppm in ^1^H-NMR spectra (Figure S7). The methyl unit -N(CH_3_)_2_·HCl appeared as a singlet at 2.94 ppm, overlapping with the methylene group in -SC*H*_2_CH_2_N-.

Then, to prepare the corresponding quaternary ammonium-functionalized dendrons, neutralization, and subsequent quaternization of -NMe_2_·HCl moieties were necessary. The first step was carried out in MeOH using aqueous aliquots of NaCO_3_ as a base (Scheme 2). Size-exclusion chromatography in acetone was selected as the purification method. Thus, dendrons EG_n_(NMe_2_)_m_ (n = 1, m = 2 (**13**); n = 2, m = 4 (**14**); n = 3, m = 8 (**15**)) were obtained as orange oils in size-dependent yields (50–80%). In the ^1^H-NMR spectra (Figure S8), all characteristic signals described for **10**–**12** were further shielded, showing that the peaks corresponding to the methylene groups overlapped around 2.50 ppm (-SiCH_2_C*H*_2_S- and -SC*H*_2_C*H*_2_N-) and that of the methyl unit (-NMe_2_) was observed at 2.22 ppm. In ^13^C-NMR spectra (Figure S9), the key peaks appeared at 27.5 (-SiCH_2_*C*H_2_S-), 29.6 (-S*C*H_2_CH_2_N-), 45.2 (-NMe_2_), and 59.1 (-SCH_2_*C*H_2_N-) ppm.

Lastly, the quaternization of amino groups (-NMe_2_) with MeI in dry THF led to the final amphiphilic cationic dendrons with vitamin E at the focal point EG_n_(NMe_3_I)_m_ (n = 1, m = 2 (**16**); n = 2, m = 4 (**17**); n = 3, m = 8 (**18**)) as orange solids with excellent yields (>90%). Compounds **16**–**18** were characterized using NMR spectroscopy. In the ^1^H NMR spectra (Figure S10), the resonances attributed to methylene groups close to the nitrogen atom were observed distinctly at 2.81 (-SiCH_2_C*H*_2_S-), 3.02 (-SC*H*_2_CH_2_N-), and 3.68 (-SCH_2_C*H*_2_N-) ppm. In addition, peaks corresponding to the methyl units (-NMe_3_I) appeared at 3.21 ppm. Analogously, resonances were shifted in the ^13^C-NMR spectra (Figure S11) with peaks corresponding to the carbon atoms in methylene and methyl fragments attached to nitrogen nuclei detected at 53.9 ppm for -NMe_3_I and 67.2 ppm for -CH_2_N-, while those bound to sulfur atoms appeared at 25.9 ppm in -SiCH_2_*C*H_2_S and 29.1 ppm in -S*C*H_2_CH_2_N-. Figure 2B shows the full structures of the amphiphilic cationic compounds **16**–**18**.

### 3.2. Micelle Formation

Our previous studies with amphiphilic carbosilane dendrons showed that the carbosilane scaffold plays an important role in the self-assembly process [33]. Specifically, the higher generation favors the micellization process. The critical micellar concentration (CMC) of amphiphilic compounds was evaluated for second- and third-generation ammonium-containing dendrons (**5**–**6** and **17**–**18**) using surface tension measurements. Due to the ionic charge of the dendrons, CMC is strongly affected by the presence of salts in the medium (see Figure S12). Salt addition decreases the repulsive electrostatic forces between headgroups and favors micelle formation. Without salt addition, data showed that no micelle formation took place below 10 mM dendron concentration. Due to the potential biomedical applications of these systems, we were interested in systems with the lowest effective concentrations. In prior reports with carbosilane dendrons having palmitic groups at the focal point, 20 mM NaCl was found to be the optimal salt concentration to achieve self-assembly [33]. Because the Cl^-^ ion could exchange with I^-^ counterion from the dendrons and interfere with the micelle formation, NaI in the same proportions was also used and evaluated to study the possible influence of counterion exchange in the aggregation process. The results indicated a higher surface tension reduction in the presence of NaI than NaCl and slightly lower surface tension with 40 mM of salt than at 20 mM of salt (Table S1). 

**Figure 3:**
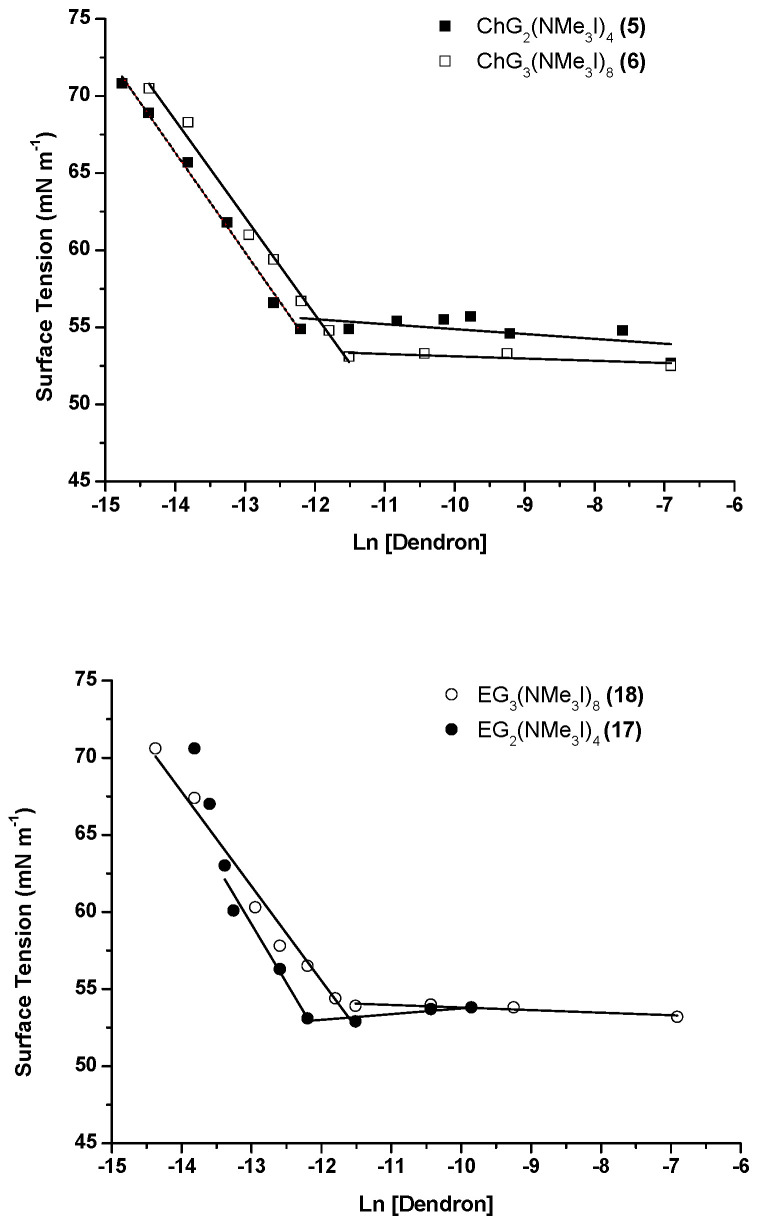
Surface tension measurements of amphiphilic dendrons with cholesterol (top; **5**–**6**) or vitamin E (bottom; **17**–**18**) at the focal point in 40 mM aqueous NaI.

CMC measurements were performed at increasing dendron concentrations in aqueous NaI solution (40 mM) at 30 °C. Figure 3 displays the surface tension curves obtained for each dendron. The CMC values listed in Table 1 range from 4.4 to 9 μM for the cationic carbosilane dendrons. 

CMC values slightly depend on the generation of the dendron but not on the focal point group. The second-generation dendrons had about two-fold-lower CMC values, implying that the second-generation dendron had a balance between lipophilicity (dendritic skeleton + tail) and hydrophilicity (number of ionic charges) that was more favorable for micelle formation. This behavior agrees with cationic bis-MPA dendrons modified with cholesterol (2.9–13 μM) [7,8] or cationic polylysine dendrons functionalized with tocopherol (1.3–15 μM) [25,26]. However, this observation is the opposite to that found for analogous cationic carbosilane dendrons with fatty acids (palmitic or hexanoic groups) at the focal point (see Table 1) [33]. The latter different profile can be attributed to the importance of a hydrophilic–lipophilic balance (HBL) in the micelle formation process of amphiphilic systems.

The Gibbs adsorption equation suitable for dilute ideal solutions can be considered for ionic micelles with an excess of salt. From the surface excess (Γ), approximate values for the area per molecule of dendrons at the air/water interface (A) can be obtained.

(1) $$\Gamma_{CMC}=-\frac{1}{RT}\frac{\partial \gamma }{\partial \ln C}$$

(2) $$\;A=\frac{1}{N\Gamma }$$

Results derived from Expressions 1 and 2 are listed in Table 1. As expected, all dendrons presented positive surface excess which reflects their preference to stay at the water/air interface instead of in the solution matrix, as expected for amphiphilic molecules. The area per molecule of dendron at the surface (A) ranged from 0.54 to 0.67 nm^2^ and is significantly lower than those found for analogous carbosilane dendrons with palmitic groups at the focal point [33]. Similar values were determined for conventional surfactants such as dodecyl trimethylammonium bromide (0.56 nm^2^ per molecule). In our case, the ionic head groups are larger in number and more charged, but we should keep in mind the presence of an excess of electrolytes. It is clear from the values in Table 1 that dendron molecules in this region of surface saturation are closely packed, with an orientation perpendicular to the surface. This fact could be explained by a more compact arrangement in the surface creating a highly dense particle compared to their palmitic analogous. Considering that the cationic head groups are the same, the cholesterol and vitamin E at the focal points favor the closest packing of head groups. 

The spontaneity of the micellar aggregation process was confirmed through Gibbs free energy calculations (ΔG^0^_mic_) based on Expression 3, in which the correction factor *i* was set up as 1 due to the saturation of the stern layer derived from the high salt concentration employed in the experiment. Table 1 shows negative ΔG^0^_mic_ values and more favorable processes in second-generation compounds, which agrees with their lower CMC data.

(3) $$\Delta_{mic}G^{0}=iRT\ln X_{CMC}$$

Hydrodynamic diameters were estimated using DLS for cationic micelles formed in saline. In the absence of drugs, the size distributions (see Figure S14) confirmed the aggregate formation. Hydrodynamic diameter values showed a decreasing tendency with their generation, contrary to the CMC trend (Table 1). These results can be explained through the dendron-tail model in which the amphiphilic balance between the dendron (polar head) and the non-polar tail, cholesterol or vitamin E in this work, controls the aggregation process. In this approach, larger polar head groups drive the formation of smaller micelles composed of fewer amphiphilic molecules. In any case, the diameters estimated using DLS are calculated considering spherical micelles. However, the values obtained are too high to be considered as spherical geometries and fit better with cylindrical micelles. The presence of NaI effectively screens the repulsive charges between the micelles, but potentially may also affect their aggregate structures. The reduction in effective head group area due to the screening of the electrostatic repulsion results in the increase in the packing geometry and the growth of cylindrical micelles. These shapes were described in our previous studies for cationic micelles prepared from carbosilane dendrons containing fatty acids at the focal point [34]. The polar head is made up of a hydrophobic carbosilane scaffold and hydrophilic terminal groups (-NMe_3_I). Consequently, the growth from G2 to G3 dendrons not only increases the hydrophobicity (skeleton) but also the hydrophilicity (number of peripheral functional groups) explaining the smaller size obtained for the highest-generation compounds. 

### 3.3. Drug Encapsulation

Drug encapsulations were performed for hydrophilic drugs including ibuprofen sodium salt, lidocaine, and procaine, as well as for the hydrophobic drug diclofenac. Only dendrons **5** and **17** were selected for these experiments based on their lowest CMC values. Both dendrons were able to load all of the drugs, although the protocols used depended on the nature of the drugs.

**Figure 4:**
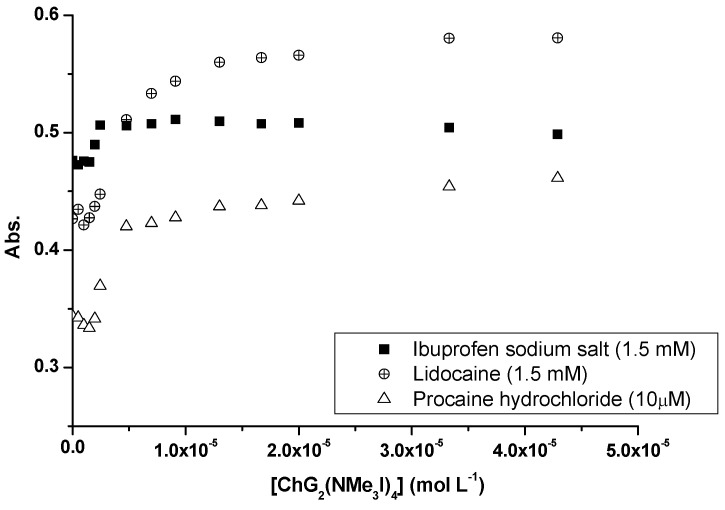

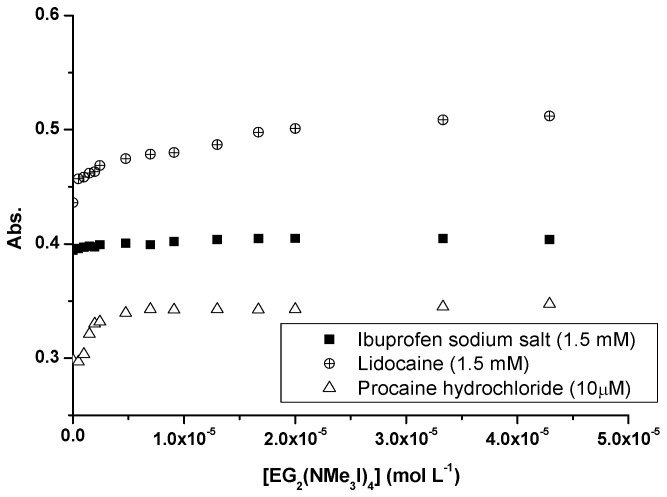
Variation of absorbance by increasing the dendron concentration at the maximum absorption wavelength for each drug.

The encapsulation of aqueous-soluble drugs was determined through the modification of their absorbance in the presence of increasing concentrations of dendrons (0.5–50 μM). For all molecules, the absorption spectra showed an increase in the drug absorbance as the dendron concentration increased (see Figure S13). This behavior indicates the association of drug molecules with dendron micelles, although drugs can be incorporated in various ways in micelles: they may locate (i) inside the hydrophobic core of the micelles, (ii) be adsorbed on the surface of micelles, or (iii), in the case of drugs with polar and non-polar regions, they may be in the polar portion between the hydrophilic peripheral groups of dendrons and the non-polar region towards the micelle core. For hydrophilic drugs such as procaine hydrochloride or hydrochlorothiazide, it is well known that a bathochromic shift is observed when transferring from water to a less polar region such as in micelles [35,36]. The absorption maxima were found at wavelengths of 272, 270, and 290 nm for ibuprofen, lidocaine, and procaine, respectively. However, no bathochromic shifts were observed. Although these behaviors suggest that the drugs were adsorbed on the micellar surface, inclusion inside of the micelle cannot be ruled out because these dendritic micelles include rather large amounts of water, disrupting the hydrophobic core [34]. The representation of absorbances at these wavelengths versus dendron concentrations showed absorbance increases for dendron concentrations below the CMC values of the corresponding dendrons (Figure 4) and suggesting that drugs affect the CMC values.

**Figure 5:**
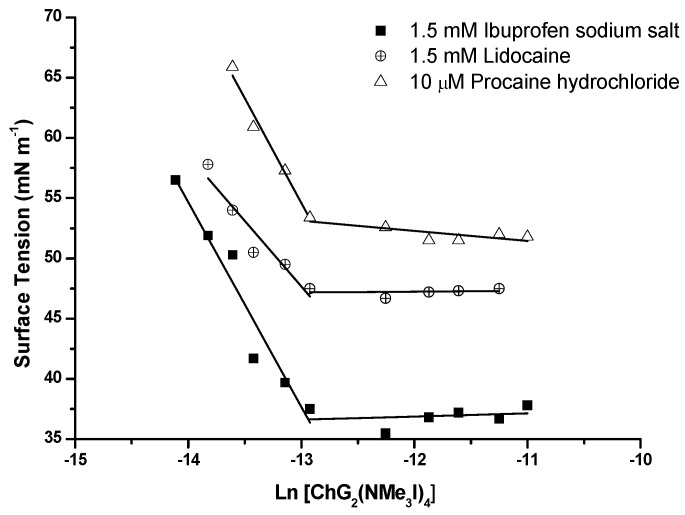

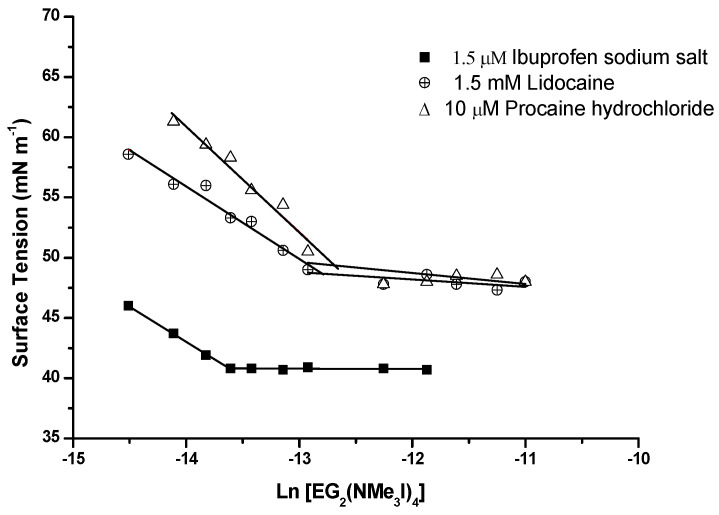
Surface tension measurements of amphiphilic dendrons with cholesterol (**top**) or vitamin E (**bottom**) at the focal point in 40 mM aqueous NaI and in the presence of drugs (■ ibuprofen ○ (1.5 mM), lidocaine (1.5 mM), or Δ procaine (10 μM).

The CMCs in the presence of drugs were measured, affording slightly lower values than those obtained in the absence of drugs (Figure 5), and confirming the influence of the drugs in the micelle formation process. The sizes of micelles were also affected by the drugs (Table 2). Particle size distributions in the presence of drugs can be seen in Figure S14. The drug incorporation increased the diameters of the micelles slightly, with the effect being least pronounced for ibuprofen and most pronounced for procaine.

From the data in Figure 4, we can observe that the difference between the absorbance of the drug completely bound to the micelle (plateau A_∞_) and the absorbance of the drug in water (A_0_) was the lowest for ibuprofen. All data considered, we can conclude that the binding affinity of ibuprofen to these dendrons is the lowest of all the drugs. 

**Figure 6:**
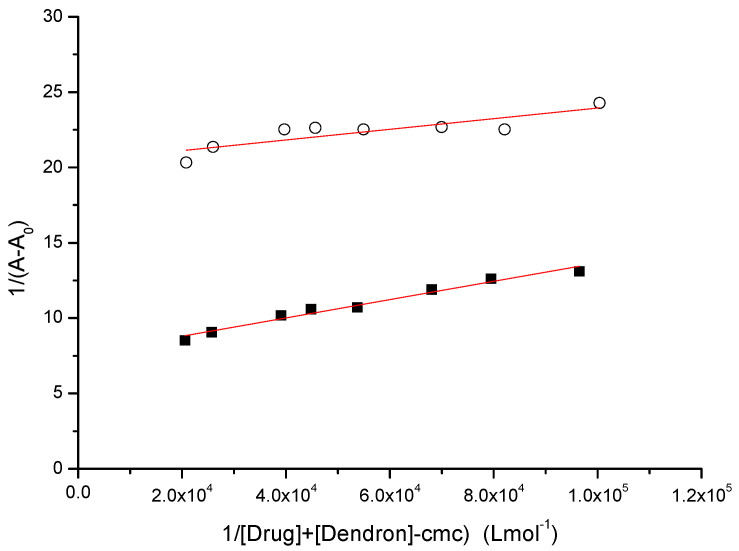
Relationship between 1/(A−A_0_) and 1/([Drug] + [Dendron] −cmc) for procaine in dendron solutions (▪ ChG_2_(NMe_3_I)_4_ (**5**); ◦ EG_2_(NMe_3_I)_4_ (**17**)).

Partition constants (*K*) were calculated for procaine using a pseudo-phase model as described in the literature [36,37]. The relationships between 1/(A−A_0_) and 1/([Drug] + [Dendron] −cmc) were not linear for lidocaine and ibuprofen. The drug concentrations for lidocaine and ibuprofen were much higher than the dendron concentration which prevents us from using this method. These high drug concentrations were necessary to obtain absorbance data. However, data obtained for procaine was the only one which could be adjusted to a linear fit (Figure 6). The log*K* values obtained were 6.7 and 7.4 for **5** and **17**, respectively. The results showed that **17** can more readily encapsulate procaine than **5**, probably due to the presence of additional hydrophobic interactions such as π-π stacking. These results suggest an improvement of more than one order of magnitude compared to other carbosilane dendritic systems previously described [31].

For hydrophobic drugs, a solubility study of diclofenac with dendrons **5** and **17** was performed, confirming the capabilities of both dendrons to enhance the solubility of diclofenac through the formation of micelles (Supporting Figure S15). The encapsulation percentage was measured using HPLC (see encapsulation protocol). Micelles were prepared with different dendron/diclofenac molar ratios, showing their optimal encapsulation values at molar ratios of 3.3:1 and 5.5:1 for dendrons **5** and **17**, respectively (Supporting Figure S16).

These micelles presented a very good encapsulation efficiency of 92% for diclofenac (Figure S16), which was the best reported in the literature so far, compared to PLGA nanoparticles (55%), nanostructured lipids (67%), or span 60/cholesterol (82.1%) [38,39,40]. It is noteworthy that in all cases reported in the literature, diclofenac sodium salt was used. The neutral form used here is more hydrophobic, which may result in higher affinity for the internal micellar domain than its ionic analogue, resulting in higher efficiencies. Diclofenac loading did not modify the hydrodynamic diameters of the micelles for cholesterol-modified dendrons while it slightly increased the diameters observed for tocopherol-modified dendrons (Table 2). The stabilities in the solution of the micellar systems loaded with diclofenac were also evaluated. Both micellar solutions displayed good colloidal stability at 25 °C in both water and PBS (10 mM), maintaining their size and the encapsulated drug for at least 7 days (Supporting Figure S17).

**Figure 7:**
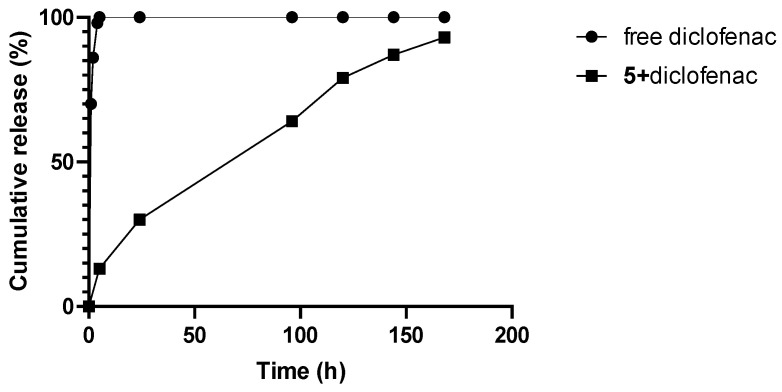
In vitro release profile of **5**+diclofenac micelles in PBS solution (pH = 7.4) at 37 °C.

We performed in vitro release studies of only dendron **5** micelles loaded with diclofenac, considering their exceptional encapsulation efficiency values achieved at lower dendron concentrations. The micelle system prepared with a **5**/diclofenac molar ratio of 3.3:1 was chosen for the study. As depicted in Figure 7, **5**+diclofenac micelles could slowly release diclofenac in a time-dependent manner, with 93% of total diclofenac released in 7 days. 

### 3.4. Toxicicy Experiments

**Figure 8:**
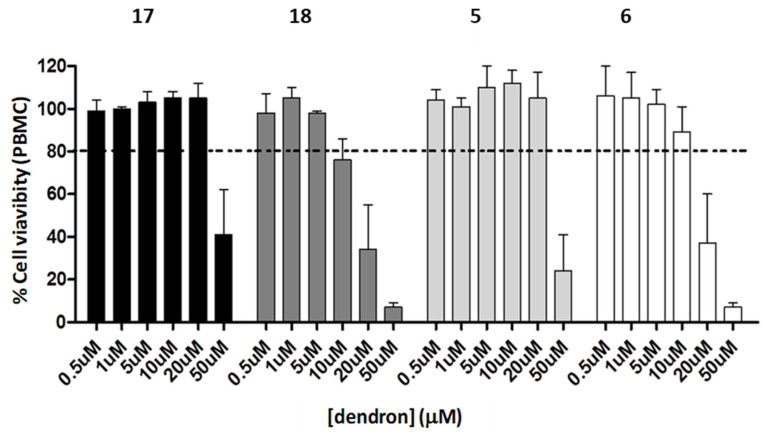
Cell viability in PBMCs of amphiphilic cationic compounds with cholesterol (**5**–**6**) or vitamin E (**17**–**18**) by MTT assay. The cells were treated with increasing concentrations of dendrons (ranged from 0.5 to 50 µM). The percent cell viability was calculated as optical density of treated/non-treated control (NT) × 100. In total 80% viability was set as the limit of toxicity. Data are represented as mean ± SD of three experiments achieved in triplicate.

The toxicity of the dendritic systems in eukaryotic cells was also studied. Safe concentrations of the dendrons were determined using MTT assay in peripheral blood mononuclear cells (PBMCs). Acceptable cellular viability was established at 80%, while lower values were considered toxic. The results are shown in Figure 8 where the maximum tolerated concentrations were 20 µM for **5** and **17**, and 10 µM for **6** and **18**. Considering that the salt concentration in the cellular medium employed is higher than the 40 mM NaI used in the CMC determination, these data reflect different generation-dependent profiles in their toxicity. Toxicity of second-generation amphiphiles **5** and **17** were independent of micelle formation, given that some assayed concentrations higher than the CMC (4.4–4.8 µM) were non-toxic. However, the toxic limit of third-generation dendrons **6** and **18** was established around the CMC values (8.0–9.0 µM) showing toxicity under the micellar regime and suggesting that a possible effect of micelle formation on toxicity could not be ruled out. The cell viability obtained for these dendrons are in the range of those measured for PAMAM-based dendrons or carbosilane dendrons containing fatty acids at the focal points [28,33,41].

## 4. Conclusions

Cationic families of dendritic amphiphiles were synthetized from cholesterol or vitamin E as hydrophobic molecules and carbosilane dendrons. Peripheral quaternary ammonium moieties were selected as the hydrophilic components of the structure and the effects of the different structures on the micellization process were examined. Second- and third-generation dendrons formed micelles, and this micellization process was favored by salt addition. Different concentrations (20 mM and 40 mM) and salt types (NaCl and NaI) were assayed, establishing the optimal conditions for aggregate formation in 40 mM NaI. The larger iodide anion (I^−^) probably produces a favorable stabilization of repulsion forces between peripheral ammonium groups. CMC values reflected a generation dependence of self-assembly, with values of approximately 5 and 10 μM for the second and third generations, respectively, and with a negligible focal point effect. Thermodynamic parameter calculations confirmed the surfactant nature of the dendritic systems and the spontaneity of the micellar process through positive values of surface excess (Γ) and negative Gibbs free energy (ΔG^0^_mic_). According to the lowest CMC values found for second-generation amphiphiles, ΔG^0^_mic_ corroborated that the micellization is energetically more favorable. Hydrophilic and hydrophobic drugs were satisfactorily loaded in micelles using different methods. The protocol employed for water-soluble drugs showed their encapsulation. This phenomenon was attributed to the reduction in CMC in the presence of the drug, which in turn resulted in a more favorable aggregation process. Partition constant (K) calculations were relevant only for procaine and showed a better encapsulation efficiency for the d-α-tocopherol modified dendron (**17**) than the cholesterol-based analogue (**5**). Regarding hydrophobic drugs, a very high diclofenac encapsulation was achieved with both cholesterol- and vitamin E-modified dendrons. The encapsulation efficiency of 92% found for diclofenac was the best described in the literature so far. In addition, DLS measurements revealed micelle size modification in the presence of hydrophilic drugs but none or very minimal change in the case of the hydrophobic ones. The toxicity of the amphiphilic systems was measured on PBMCs, showing that, unlike the third-generation system, the second-generation system was not toxic until 20 μM, which is above their CMC values, opening a window for their use in a micellar regimen where they can operate as drug delivery systems for different biomedical applications.

## Figures and Tables

**Table 1:** CMC and diameter values of amphiphilic dendrons with cholesterol (**5**–**6**) or vitamin E (**17**–**18**) at the focal point in 40 mM aqueous NaI and comparison with values of analogous previously published systems.

Dendron	CMC (μM)	Diameter (nm)	Γ (µmol/m^2^)	A (nm^2^/Molecule)	ΔG^0^_mic_ (kJ/mol)
ChG_2_(NMe_3_I)_4_ (**5**)	4.4 ± 0.7	9.2 ± 0.4	2.6	0.64	−40.5
ChG_3_(NMe_3_I)_8_ (**6**)	9.0 ± 1.0	6.6 ± 0.1	2.5	0.65	−38.7
EG_2_(NMe_3_I)_4_ (**17**)	5.1 ± 0.9	7.7 ± 0.2	3.1	0.54	−40.1
EG_3_(NMe_3_I)_8_ (**18**)	8.0 ± 1.0	6.5 ± 0.3	2.5	0.67	−39.1
* C_16_G_2_(NMe_3_I)_4_	19 ± 1	5.6 ± 0.1	0.77	2.1	-
* C_16_G_3_(NMe_3_I)_8_	17 ± 1	5.6 ± 0.1	0.69	2.4	-
* C_6_G_3_(NMe_3_I)_8_	12 ± 1	3.8 ± 0.3	0,83	2.0	-

* Data obtained from Ref. [33].

**Table 2:** CMC and diameter values of dendrons with cholesterol (**5**) or vitamin E (**17**) at the focal point obtained in absence or presence of ibuprofen (1.5 mM), lidocaine (1.5 mM), or procaine (10 μM).

Dendron	CMC (μM)	Diameter (nm)	Dendron	CMC (μM)	Diameter (nm)
ChG_2_(NMe_3_I)_4_ (**5**)	4.4 ± 0.7	9.2 ± 0.4	EG_2_(NMe_3_I)_4_ (**17**)	4.8 ± 0.9	7.7 ± 0.2
**5** + ibuprofen	2.4 ± 0.2	10.0 ± 0.6	**17** + ibuprofen	1.22 ± 0.02	10.7 ± 0.4
**5** + lidocaine	2.4 ± 0.3	11.9 ± 0.8	**17** + lidocaine	3.0 ± 0.4	11.1 ± 0.2
**5** + procaine	2.6 ± 0.2	12.9 ± 0.3	**17** + procaine	3.7 ± 0.5	11.0 ± 0.7
**5** + diclofenac	-	9.1 ± 0.2	**17** + diclofenac	-	8.9 ± 0.2

## Data Availability

The data presented in this study are available in this article (and Supplementary Materials).

## Supplementary Materials

The following supporting information can be downloaded at: https://www.mdpi.com/article/10.3390/pharmaceutics16040451/s1, Table S1. Surface tension values of 1 mM solution of dendrons **5** and **17** at different concentrations of NaCl and NaI. Figure S1. ^1^H-NMR spectrum of dendron ChG_1_(SNMe_2_)_2_ (**1**) in CDCl_3_. Figure S2. ^13^C-NMR spectrum of dendron ChG_1_(SNMe_2_)_2_ (**1**) in CDCl_3_. Figure S3. ^1^H-NMR spectrum of dendron ChG_1_(SNMe_3_I)_2_ (**4**) in CD_3_OD. Figure S4. ^13^C-NMR spectrum of dendron ChG_1_(SNMe_3_I)_2_ (**4**) in CD_3_OD. Figure S5. ^1^H-NMR spectrum of dendron EG_1_(V)_2_ (**7**) in CDCl_3_. Figure S6. ^13^C-NMR spectrum of dendron EG_1_(V)_2_ (**7**) in CDCl_3_. Figure S7. ^1^H-NMR spectrum of dendron EG_1_(SNMe_2_·HCl)_2_ (**10**) in CD_3_OD. Figure S8. ^1^H-NMR spectrum of dendron EG_1_(SNMe_2_)_2_ (**13**) in CDCl_3_. Figure S9. ^13^C-NMR spectrum of dendron EG_1_(SNMe_2_)_2_ (**13**) in CDCl_3_. Figure S10. ^1^H-NMR spectrum of dendron EG_1_(SNMe_3_I)_2_ (**16**) in CD_3_OD. Figure S11. ^13^C-NMR spectrum of dendron EG1(SNMe_3_I)_2_ (**16**) in CD_3_OD. Figure S12. Surface tension measurements of dendrons ChG_2_(SNMe_3_I)_4_ (**5**) and EG_2_(SNMe_3_I)_4_ (**17**) at increasing concentrations without the presence of salt. Figure S13. UV-Vis absorption spectra of saline solution (40 mM of NaI) of increasing concentrations of dendrons **5** and **17** in presence of ibuprofen (1.5 mM), procaine (1.5 mM) or lidocaine (10 μM). Figure S14. Particle size distribution of amphiphilic dendrons **5** and **17** in absence or presence of ibuprofen (1.5 mM), lidocaine (1.5 mM) or procaine (10 μM) with 40 mM of NaI. Figure S15. Solubilization of diclofenac by dendron **5** or **17**. Figure S16. Relation between dendron **5** or **17** and encapsulation percentage. Optime dendron:diclofenac molar ratio 3.3:1 (**5**) and 5.5:1 (**17**). Figure S17. Time-dependent hydrodynamic size and % diclofenac encapsulated change **5**+diclofenac and **17**+diclofenac nanomicelles dispersed in water or PBS (10 mM).
