# Supplementary material for: Carbosilane Dendritic Amphiphiles from Cholesterol or Vitamin E for Micelle Formation
Source: Pharmaceutics. 2024 Mar 25;16(4):451. doi: 10.3390/pharmaceutics16040451 (PMC11053416; doi:10.3390/pharmaceutics16040451)
Supplement: Supplementary file 1 [file pharmaceutics-16-00451-s001.zip › pharmaceutics-2800220-supplementary.pdf]

SUPPORTING INFORMATION

Regular Article

Carbosilane Dendritic Amphiphiles from Cholesterol or Vitamin E for Micelle Formation

Gabriel Mencia [1,2,3], Sergio Algar [1,2], Tania Lozano-Cruz [1,2,3], Mª Ángeles Muñoz-Fernández [2,4,5], Elizabeth R. Gillies [6], Jesús Cano [1,2,3], Mercedes Valiente [7,*] and Rafael Gómez [1,2,3,*]

1. Department of Organic and Inorganic Chemistry, Research Institute in Chemistry "Andrés M. Del Río" (IQAR), University of Alcalá, 28805 Madrid, Spain; gabi.men.ber@gmail.com (G.M.); sergioalgar@ucm.es (S.A.); tania.lozano@uah.es (T.L.-C.); jesus.cano@uah.es (J.C.)
2. Networking Research Center on Bioengineering, Biomaterials and Nanomedicine (CIBER-BBN), 28029 Madrid, Spain; mmunoz.hgugm@gmail.com
3. Ramón y Cajal Health Research Institute (IRYCIS), 28034 Madrid, Spain
4. Laboratory Platform (Immunology), General Universitary Hospital Gregorio Marañón (HGUGM), 28007 Madrid, Spain
5. Spanish HIV HGM BioBank, Health Research Institute Gregorio Marañón (HGUGM), 28007 Madrid, Spain
6. Department of Chemistry and Chemical and Biochemical Engineering, School of Biomedical Engineering, University of Western Ontario, London, ON N6G1Z1, Canada; egillie@uwo.ca
7. Department of Analytical Chemistry, Physical Chemistry and Chemical Engineering, Research Institute in Chemistry "Andrés M. Del Río" (IQAR), University of Alcalá, 28805 Madrid, Spain

* Correspondence: mercedes.valiente@uah.es (M.V.); rafael.gomez@uah.es (R.G.); Tel.: +34-91-8854670 (M.V.); +34-91-8854685 (R.G.)

Table of contents

Figure S1. ^{1}H-NMR spectrum of dendron $ChG_1(SNMe_2)_2$ (**1**) in $CDCl_3$ 10

Figure S2. ^{13}C-NMR spectrum of dendron $ChG_1(SNMe_2)_2$ (**1**) in $CDCl_3$ 10

Figure S3. ^{1}H-NMR spectrum of dendron $ChG_1(SNMe_3I)_2$ (**4**) in CD_3OD 11

Figure S4. ^{13}C-NMR spectrum of dendron $ChG_1(SNMe_3I)_2$ (**4**) in CD_3OD 11

Figure S5. ^{1}H-NMR spectrum of dendron $EG_1(V)_2$ (**7**) in $CDCl_3$ 12

Figure S6. ^{13}C-NMR spectrum of dendron $EG_1(V)_2$ (**7**) in $CDCl_3$ 12

Figure S7. ^{1}H-NMR spectrum of dendron $EG_1(SNMe_2{\cdot}HCl)_2$ (**10**) in CD_3OD 13

Figure S8. ^{1}H-NMR spectrum of dendron $EG_1(SNMe_2)_2$ (**13**) in $CDCl_3$ 13

Figure S9. ^{13}C-NMR spectrum of dendron $EG_1(SNMe_2)_2$ (**13**) in $CDCl_3$ 14

Figure S10. ^{1}H-NMR spectrum of dendron $EG_1(SNMe_3I)_2$ (**16**) in CD_3OD 14

Figure S11. ^{13}C-NMR spectrum of dendron $EG_1(SNMe_3I)_2$ (**16**) in CD_3OD 15

Figure S12. Surface tension measurements of dendrons $ChG_2(SNMe_3I)_4$ (**5**) and $EG_2(SNMe_3I)_4$ (**17**) at increasing concentrations without the presence of salt 15

Table S1. Surface tension values of 1 mM solution of dendrons **5** and **17** at different concentrations of NaCl and NaI 16

Figure S13. UV-Vis absorption spectra of saline solution (40 mM of NaI) of increasing concentrations of dendrons **5** and **17** in presence of ibuprofen (1.5 mM), procaine (1.5 mM) or lidocaine (10 μM). 16

Figure S14. Particle size distribution of amphiphilic dendrons **5** and **17** in absence or presence of ibuprofen (1.5 mM), lidocaine (1.5 mM) or procaine (10 μM) with 40 mM of NaI. 17

Figure S15. Solubilization of diclofenac by dendron **5** or **17.** 18

Figure S16. Relation between dendron **5** or **17** and encapsulation percentage. Optime dendron:diclofenac molar ratio 3.3:1 (**5**) and 5.5:1 (**17**) 18

Figure S17. Time-dependent hydrodynamic size and % diclofenac encapsulated change **5**+diclofenac and **17**+diclofenac nanomicelles dispersed in water or PBS (10 mM). 19

Synthesis and characterization

Synthesis of $ChG_2(NMe_2)_4$ (2).

Following the procedure described for **1**, dendron **2** was obtained as white solid (71%) using $NH_2G_2(SNMe_2)_4$ (**ii**) (1.7 g, 1.24 mmol),[1] cholesteryl chloroformate (0.556 g; 1.24 mmol) and NEt_3 (0.21 mL; 1.5 mmol).

^{1}H-NMR ($CDCl_3$): δ (ppm) -0.07 (s, 3H, $-Si(CH_3)-$), -0.04 (s, 6H, $-Si(CH_3)-$), 0.45-0.62 (m, 18H, $-NCH_2CH_2CH_2CH_2Si-$, $-SiCH_2CH_2CH_2Si-$, $-SiCH_2CH_2S-$), 0,65 (s, 3H, CH_3C-, cholesterol), 0.81 (d, 6H, CH_3CH-, cholesterol), 0.84 (d, 3H, CH_3CH-, cholesterol), 0.98 (s, 3H, CH_3C-, cholesterol), 0,91-2,02 (m, 36H, $-CH_2-$, $-CH-$, cholesterol, $-NCH_2CH_2CH_2CH_2Si-$, $-SiCH_2CH_2CH_2Si-$), 2.18 (m, 24H, $-N(CH_3)_2$), 2.51 (m, 24H, $-SiCH_2CH_2S-$, $-SCH_2CH_2N-$, $-SCH_2CH_2N-$), 3.12 (m, 2H, $-NCH_2CH_2CH_2CH_2Si-$), 4.49 (m, 1H, $-CHCONH-$), 4.76 (m, 1H, $-CONH-$), 5.30 (m, 1H, $-C=CHCH_2-$, cholesterol). **^{13}C-NMR** ($CDCl_3$): δ (ppm) -5.35 ($-Si(CH_3)-$), -5.30 ($-Si(CH_3)-$), 13.8-14.4 ($-SiCH_2-$), 11.7, 18.6, 19.2, 21.1, 22.5, 22.9, 23.5, 24.3, 27.9, 28.1, 31.8, 35.7, 35.9, 36.3, 36.9, 38.7, 39.4, 39.7, 42.2, 50.1, 56.2, 56.6 (CH_3-, $-CH_2-$, $-CH-$, $-C-$, cholesterol), 20.6 ($-NCH_2CH_2CH_2CH_2Si-$), 21,3 ($-SiCH_2CH_2CH_2Si-$), 27.4 ($-SiCH_2CH_2S-$), 29.7 ($-SCH_2CH_2N-$), 34.3 ($-NCH_2CH_2CH_2CH_2Si-$), 40.7 ($-NCH_2CH_2CH_2CH_2Si-$), 45.1 ($-N(CH_3)_2$), 58.7 ($-SCH_2CH_2N-$), 74.4 ($-CHCONH-$), 122.1 ($-C=CHCH_2-$, cholesterol), 139.2 ($-C=CHCH_2-$, cholesterol), 156.2 ($-CONH-$). **Mass spectroscopy**: $[M+H]^+$ = 1226.8584 Da (calcd. = 1226.8566 Da). **Elemental analysis** $C_{65}H_{131}N_5O_2S_4Si_3$ (1227.29 g/mol): calcd. = C, 63.61; H, 10.76; N, 5.71; O, 2.61; S, 10.45; Si, 6.87. Found = C, 64.18; H, 11.02; N, 4.92; S, 10.11.

Synthesis of $ChG_3(NMe_2)_8$ (3).

Following the procedure described for **1**, dendron **3** was obtained as white solid (82%) using $NH_2G_3(SNMe_2)_8$ (**iii**) (1.9 g, 0.80 mmol),[1] cholesteryl chloroformate (0.36 g; 0.80 mmol) and NEt_3 (0.21 mL; 1.5 mmol).

^{1}H-NMR ($CDCl_3$): δ (ppm) -0.08 (s, 9H, $-Si(CH_3)-$), 0.02 (s, 12H, $-Si(CH_3)-$), 0.46-0.64 (m, 42H, $-NCH_2CH_2CH_2CH_2Si-$, $-SiCH_2CH_2CH_2Si-$, $-SiCH_2CH_2S-$), 0.67 (s, 3H, CH_3C-, cholesterol), 0.85 (d, 6H, CH_3CH-, cholesterol), 0.91 (d, 3H, CH_3CH-, cholesterol), 1.00 (s, 3H, CH_3C-, cholesterol), 0.88-2.04 (m, 44H, $-CH_2-$, $-CH-$, cholesterol, $-NCH_2CH_2CH_2CH_2Si-$, $-SiCH_2CH_2CH_2Si-$), 2.25 (m, 48H, $-N(CH_3)_2$), 2.55 (m, 48H, $-SiCH_2CH_2S-$, $-SCH_2CH_2N-$, $-SCH_2CH_2N-$), 3.13 (m, 2H, $-NCH_2CH_2CH_2CH_2Si-$), 4.47 (m, 1H, $-CHCONH-$), 4.90 (m, 1H, $-CONH-$), 5.35 (m, 1H, $-C=CHCH_2-$, cholesterol). **^{13}C-NMR** ($CDCl_3$): δ

(ppm) -5.38 (-Si(CH_3)-), -5.30 (-Si(CH_3)-), 13.8 (-$NCH_2CH_2CH_2CH_2Si$-), 11.8, 18.7, 19.3, 21.0, 22.4, 22.8, 23.7, 24.2, 27.7, 28.0, 31.8, 35.4, 36.0, 36.1, 37.0, 38.5, 39.2, 39.7, 42.2, 49.9, 56.2, 56.7 (CH_3-, -CH_2-, -CH-, -C-, cholesterol), 14.2-14.6 (-$SiCH_2CH_2CH_2Si$-), 20.7 (-$NCH_2CH_2CH_2CH_2Si$-), 21.4-21.8 (-$SiCH_2CH_2CH_2Si$-), 27.4 (-$SiCH_2CH_2S$-), 30.0 (-SCH_2CH_2N-), 34.6 (-$NCH_2CH_2CH_2CH_2Si$-), 40.5 (-$NCH_2CH_2CH_2CH_2Si$-), 45.7 (-N(CH_3)$_2$), 59.1 (-SCH_2CH_2N-), 74.1 (-CHCONH-), 122.4 (-C=$CHCH_2$-, cholesterol), 139.3 (-*C*=$CHCH_2$-, cholesterol), 155.9 (-*C*ONH-). **Mass spectroscopy**: $[M+H]^+$ = 2095.3793 Da (calcd. = 2095.3848 Da). **Elemental analysis** $C_{105}H_{223}N_9O_2S_8Si_7$ (2097.08 g/mol): calcd. = C, 60.14; H, 10.72; N, 6.01; O, 1.53; S, 12.23; Si, 9.37. Found = C, 59.28; H, 10.54; N, 5.63; S, 11.88.

Synthesis of ChG$_2$(NMe$_3$I)$_4$ (5).

Following the procedure described for **4**, dendron **5** was obtained as white solid (97%) using ChG$_2$(NMe$_2$)$_4$ (**2**) (0.50 g, 0.41 mmol), MeI (0.15 mL, 2.41 mmol).

^{1}H-NMR (D_2O): δ (ppm) 0.12 (s, 9H, -Si(C*H*$_3$)-), 0.58-0.72 (m, 18H, -$NCH_2CH_2CH_2CH_2Si$-, -$SiCH_2CH_2CH_2Si$-, -$SiCH_2CH_2S$-), 0.74 (s, 3H, C*H*$_3$C-, cholesterol), 0,89 (d, 6H, C*H*$_3$CH-, cholesterol), 0.93 (d, 3H, C*H*$_3$CH-, cholesterol), 1.10 (s, 3H, C*H*$_3$C-, cholesterol), 0.90-2.38 (m, 36H, -C*H*$_2$-, -C*H*-, cholesterol, -$NCH_2CH_2CH_2CH_2Si$-, -$SiCH_2CH_2CH_2Si$-), 2.71 (m, 8H, -$SiCH_2CH_2S$-), 2.96 (m, 8H, -SCH_2CH_2N-), 3.14 (m, 2H, -$NCH_2CH_2CH_2CH_2Si$-), 3.18 (s, 36H, -N(C*H*$_3$)$_3$), 3.66 (m, 8H, -SCH_2CH_2N-), 4.42 (m, 1H, -C*H*CONH-), 5.38 (m, 1H, -C=C*H*CH_2-, cholesterol). **^{13}C-NMR** (D_2O): δ (ppm) -5.03 (-Si(CH_3)-), -5.12 (-Si(CH_3)-), 14.4-15.7 (-$SiCH_2$-), 12.3, 19.5, 22.5, 23.1, 23.4, 24.9, 25.6, 29.1, 29.2, 29.5, 33.1, 33.5, 37.0, 37.2, 37.7, 38.1, 39.5, 40.7, 43.4, 51.3, 57.5, 58.2 (CH_3-, -CH_2-, -CH-, -C-, cholesterol), 21.9 (-$NCH_2CH_2CH_2CH_2Si$-), 22.2 (-$SiCH_2CH_2CH_2Si$-), 32.6 (-$SiCH_2CH_2S$-), 34.5 (-$NCH_2CH_2CH_2CH_2Si$-), 38.7 (-SCH_2CH_2N-), 41.4 (-$NCH_2CH_2CH_2CH_2Si$-), 54.2 (-N(CH_3)$_3$), 66.8 (-SCH_2CH_2N-), 74.3 (-CHCONH-), 122.2 (-C=$CHCH_2$-, cholesterol), 140.9 (-*C*=$CHCH_2$-, cholesterol), 158.4 (-CONH-). **Mass spectroscopy**: $[M-I]^+$ = 1666.6588 Da (calcd. = 1666.6566 Da). **Elemental analysis** $C_{69}H_{143}I_4N_5O_2S_4Si_3$ (1795.05 g/mol): calcd. = C, 46.17; H, 8.03; I, 28.28; N, 3.90; O, 1.78; S, 7.14; Si, 4.69. Found = C, 45.95; H, 7.97; N, 3.67; S, 6.83.

Synthesis of ChG$_3$(NMe$_3$I)$_8$ (6).

Following the procedure described for **4**, dendron **6** was obtained as white solid (98%) using ChG$_3$(NMe$_2$)$_8$ (**3**) (0.50 g, 0.24 mmol), MeI (0.15 mL, 2.41 mmol).

^{1}H-NMR (D_2O): δ (ppm) 0.09 (s, 21H, -Si(CH_3)-), 0.60-0.75 (m, 42H, -$NCH_2CH_2CH_2CH_2$Si-, -SiCH_2CH_2CH_2Si-, -SiCH_2CH_2S-), 0.76 (s, 3H, CH_3C-, cholesterol), 0.93 (d, 6H, CH_3CH-, cholesterol), 0.98 (d, 3H, CH_3CH-, cholesterol), 1.07 (s, 3H, CH_3C-, cholesterol), 0.94-2.00 (m, 44H, -CH_2-, -CH-, cholesterol, -$NCH_2CH_2CH_2CH_2$Si-, -SiCH_2CH_2CH_2Si-), 2.74 (m, 16H, -SiCH_2CH_2S-), 3.00 (m, 16H, -SCH_2CH_2N-), 3.11 (m, 2H, -$NCH_2CH_2CH_2CH_2$Si-), 3.22 (m, 72H, -N(CH_3)$_3$), 3.71 (m, 16H, -SCH_2CH_2N-), 4.39 (m, 1H, -CHCONH-), 5.42 (m, 1H, -C=CHCH_2-, cholesterol). **^{13}C-NMR** (D_2O): δ (ppm) -4.98 (-Si(CH_3)-), 14.1-15.5 (-SiCH_2-), 12.3, 19.5, 22.8, 23.2, 23.4, 24.9, 25.5, 29.1, 29.4, 29.8, 33.3, 33.4, 36.8, 37.3, 37.5, 37.9, 39.8, 409, 43,3, 51,3, 57,8, 58,5 (CH_3-, -CH_2-, -CH-, -C-, cholesterol), 22,1 (-$NCH_2CH_2CH_2CH_2$Si-), 22.2-22.5 (-SiCH_2CH_2CH_2Si-), 32.5 (-SiCH_2CH_2S-), 34.7 (-$NCH_2CH_2CH_2CH_2$Si-), 38.3 (-SCH_2CH_2N-), 41.1 (-$NCH_2CH_2CH_2CH_2$Si-), 54.6 (-N(CH_3)$_2$), 67.7 (-SCH_2CH_2N-), 74.2 (-CHCONH-), 121.7 (-C=CHCH_2-, cholesterol), 140.1 (-C=CHCH_2-, cholesterol), 157.9 (-CONH-). **Mass spectroscopy**: [cholesterol+H]$^+$ = 387.3625 Da (calcd. = 387.3621 Da); [M-I-ChCOO]$^+$ = 2690.5593 Da (calcd. = 2690.5625 Da). **Elemental analysis** $C_{113}H_{247}I_8N_9O_2S_8Si_7$ (3232.59 g/mol): calcd. = C, 41.99; H, 7.70; I, 31.41; N, 3.90; O, 0.99; S, 7.93; Si, 6.08. Found = C, 41.64; H, 7.41; N, 3.45; S, 5.63.

Synthesis of $EG_2(V)_4$ (8).

Following a similar procedure described for **7**, dendron **8** was obtained as orange oil (88%) using $BrG_2(V)_4$ (**v**)[1] (1.0 g; 2.18 mmol), d-α-tocopherol (0.940 g; 2.18 mmol), K_2CO_3 (0.614 g; 4.44 mmol), crown ether 18-6 (0.058 g; 0.22 mmol).

^{1}H-NMR ($CDCl_3$): δ (ppm) -0.05 (s, 3H, -Si(CH_3)-), 0.14 (s, 6H, -Si(CH_3)-), 0.59 (m, 6H, -$OCH_2CH_2CH_2CH_2$Si-, -SiCH_2CH_2CH_2SiVinilo-), 0.75 (m, 4H, -CH_2SiVinilo-), 0.87 (m, 12H, CH_3CH-), 1.00-1.66 (m, 27H, -CH_2-, -CH-, tocopherol, -$OCH_2CH_2CH_2CH_2$Si-, -SiCH_2CH_2CH_2Si-), 1.24 (s, 3H, -CH_2C(CH_3)OC$_{Ar}$-), 1.80 (m, 4H, -CH_2C(CH_3)OC$_{Ar}$-, -$OCH_2CH_2CH_2CH_2$Si-), 2.09 (s, 3H, CH_3C$_{Ar}$-), 2.13 (s, 3H, CH_3C$_{Ar}$-), 2.17 (s, 3H, CH_3C$_{Ar}$-), 2.57 (t, 2H, -C$_{Ar}$CH_2CH_2-), 3.63 (t, 2H, -$OCH_2CH_2CH_2CH_2$Si-), 5.70 (m, 4H, -SiCH=CH_2), 6.04 (m, 8H, -SiCH=CH_2). **^{13}C-NMR** ($CDCl_3$): δ (ppm) -5.38 (-Si(CH_3)-), -5.24 (-Si(CH_3)-), 11.8, 12.0, 12.7 (CH_3C$_{Ar}$-), 13.8-14.2 (-SiCH_2-), 19.4, 19.8, 20.6, 20.9, 22.6, 22.8, 23.8, 24.7, 25.0, 28.2, 31.1, 32.4, 32.7, 37.4, 37.6, 39.4, 40.3 (-CH_3-, -CH_2-, -CH-, tocopherol), 21.4 (-SiCH_2CH_2CH_2Si-), 29.8 (-$OCH_2CH_2CH_2CH_2$Si-), 34.3 (-$OCH_2CH_2CH_2CH_2$Si-), 72.9 (-$OCH_2CH_2CH_2CH_2$Si-), 74.5 (-CH_2(CH_3)COC$_{Ar}$-), 117.1 (-C$_{Ar}$CH_2CH_2-), 122.8, 125.5, 127.4 (CH_3C$_{Ar}$-), 132.7 (-SiCH=CH_2), 136.6 (-SiCH=CH_2), 147.6 (-C$_{Ar}$OC-), 148.1 ((-C$_{Ar}$OCH_2-). **Mass spectroscopy**: [M+H]$^+$ = 807.6294 Da (calcd. =

807.6321 Da). **Elemental análisis** $C_{50}H_{90}O_2Si_3$ (807.52 g/mol): calcd. = C, 74.37; H, 11.23; O, 3.96; Si, 10.43. Found = C, 73.71; H, 10.83.

Synthesis of $EG_3(V)_8$ (9).

Following a similar procedure described for **7**, dendron **9** was obtained as orange oil (93%) using $BrG_3(V)_8$ (**vi**)[1] (1.0 g; 1.10 mmol), d-α-tocopherol (0.475 g; 1.10 mmol), K_2CO_3 (0.310 g; 2.24 mmol), crown ether 18-6 (0.030 g; 0.11 mmol).

^{1}H-NMR ($CDCl_3$): δ (ppm) -0.07 (s, 9H, -Si(CH_3)-), 0.11 (s, 12H, -Si(CH_3)-), 0.58-0.79 (m, 26H, -$OCH_2CH_2CH_2CH_2Si$-, -$SiCH_2CH_2CH_2Si$-), 0.89 (m, 12H, CH_3CH-), 1.00-1.69 (m, 35H, -CH_2-, -CH-, tocopherol, -$OCH_2CH_2CH_2CH_2Si$-, -$SiCH_2CH_2CH_2Si$-), 1.27 (s, 3H, -$CH_2C(CH_3)OC_{Ar}$-), 1.83 (m, 4H, -$CH_2C(CH_3)OC_{Ar}$-, -$OCH_2CH_2CH_2CH_2Si$-), 2.11 (s, 3H, CH_3C_{Ar}-), 2.14 (s, 3H, CH_3C_{Ar}-), 2.18 (s, 3H, CH_3C_{Ar}-), 2.56 (t, 2H, -$C_{Ar}CH_2CH_2$-), 3.68 (t, 2H, -$OCH_2CH_2CH_2CH_2Si$-), 5.72 (m, 8H, -$SiCH{=}CH_2$), 6.08 (m, 16H, -$SiCH{=}CH_2$). **^{13}C-NMR** ($CDCl_3$): δ (ppm) -5.36 (-Si(CH_3)-), -5.21 (-Si(CH_3)-), 11.9, 12.2, 12.7 (CH_3C_{Ar}-), 13.6-14.2 (-$SiCH_2$-), 19.2, 19.7, 20.6, 20.8, 22.7, 22., 23.7, 24.6, 24.8, 28.1, 31.2, 32.4, 32.6, 37.3, 37.5, 39.3, 40.5 (-CH_3-, -CH_2-, -CH-, tocopherol), 21.4-21.6 (-$SiCH_2CH_2CH_2Si$-), 29.7 (-$OCH_2CH_2CH_2CH_2Si$-), 33.9 (-$OCH_2CH_2CH_2CH_2Si$-), 73.0 (-$OCH_2CH_2CH_2CH_2Si$-), 74.1 (-$CH_2(CH_3)COC_{Ar}$-), 117.6 (-$C_{Ar}CH_2CH_2$-), 122.8, 125.3, 127.6 (CH_3C_{Ar}-), 132.9 (-$SiCH{=}CH_2$), 136.5 (-$SiCH{=}CH_2$), 147.8 (-$C_{Ar}OC$-), 148.1 ((-$C_{Ar}OCH_2$-). **Mass spectroscopysas**: $[M+H]^+$ = 1255.9128 Da (calcd. = 1255.9154 Da). **Elemental analysis** $C_{74}H_{138}O_2Si_7$ (1256.51 g/mol): calcd. = C, 70.74; H, 11.07; O, 2.55; Si, 15.65. Found = C, 70.12; H, 10.69.

Synthesis of $EG_2(SNMe_2{\cdot}HCl)_4$ (11).

Following a similar procedure described for **10**, dendron **11** was obtained as white solid (95%) using $EG_2(V)_4$ (**8**) (1.0 g; 1.24 mmol), $SH(CH_2)_2SNMe_2{\cdot}HCl$ (0.738 g; 5.21 mmol), DMPA (0.073 g; 0.28 mmol).

^{1}H-NMR (CD_3OD): δ (ppm) 0.02 (s, 3H, -Si(CH_3)-), 0.13 (s, 6H, -Si(CH_3)-), 0.66-0.80 (m, 18H, -$OCH_2CH_2CH_2CH_2Si$-, -$SiCH_2$-), 0.85 (m, 12H, CH_3CH-), 0.95-1.65 (m, 27H, -CH_2-, -CH-, tocopherol, -$OCH_2CH_2CH_2CH_2Si$-, -$SiCH_2CH_2CH_2Si$-), 1.24 (s, 3H, -$CH_2C(CH_3)OC_{Ar}$-), 1.83 (m, 4H, -$CH_2C(CH_3)OC_{Ar}$-, -$OCH_2CH_2CH_2CH_2Si$-), 1.98-2.17 (m, 9H, CH_3C_{Ar}-), 2.54 (t, 2H, -$C_{Ar}CH_2CH_2$-), 2.78 (m, 8H, -$SiCH_2CH_2S$-), 2.91 (m, 24H, -$N(CH_3)_2HCl$), 3.00 (m, 8H, -SCH_2CH_2N-), 3.38 (m, 8H, -SCH_2CH_2N-), 3.63 (t, 2H, -$OCH_2CH_2CH_2CH_2Si$-).

Synthesis of $EG_3(SNMe_2{\cdot}HCl)_8$ (12).

Following a similar procedure described for **10**, dendron **12** was obtained as white solid (95%) using $EG_3(V)_8$ (**9**) (1.0 g; 0.80 mmol), $SH(CH_2)_2SNMe_2{\cdot}HCl$ (0.985 g; 6.95 mmol), DMPA (0.102 g; 0.40 mmol). **^{1}H-NMR** (CD_3OD): δ (ppm) 0.05 (s, 9H, -Si(C*H*$_3$)-), 0.13 (s, 12H, -Si(C*H*$_3$)-), 0.65-0.84 (m, 42H, -OCH$_2$CH$_2$CH$_2$C*H*$_2$Si-, -SiC*H*$_2$-), 0.87 (m, 12H, C*H*$_3$CH-), 0.95-1.65 (m, 35H, -C*H*$_2$-, -C*H*-, tocopherol, -OCH$_2$CH$_2$C*H*$_2$CH$_2$Si-, -SiCH$_2$C*H*$_2$CH$_2$Si-), 1.21 (s, 3H, -CH$_2$C(C*H*$_3$)OC$_{Ar}$-), 1.82 (m, 4H, -C*H*$_2$C(CH$_3$)OC$_{Ar}$-, -OCH$_2$C*H*$_2$CH$_2$CH$_2$Si-), 1.98-2.21 (m, 9H, C*H*$_3$C$_{Ar}$-), 2.56 (t, 2H, -C$_{Ar}$C*H*$_2$CH$_2$-), 2.72 (m, 16H, -SiCH$_2$C*H*$_2$S-), 2.92 (m, 48H, -N(C*H*$_3$)$_2$HCl), 3.02 (m, 16H, -SC*H*$_2$CH$_2$N-), 3.42 (m, 16H, -SCH$_2$C*H*$_2$N-), 3.66 (t, 2H, -OC*H*$_2$CH$_2$CH$_2$CH$_2$Si-).

Synthesis of $EG_2(SNMe_2)_4$ (14).

Following a similar procedure described for **13**, dendron **14** was obtained as yellow oil (78%) using $EG_2(SNMe_2{\cdot}HCl)_4$ (**11**) (1.7 g; 1.24 mmol), Na_2CO_3 (0.817 g; 7.71 mmol).

^{1}H-NMR ($CDCl_3$): δ (ppm) -0.08 (s, 3H, -Si(C*H*$_3$)-), -0.01 (s, 6H, -Si(C*H*$_3$)-), 0.47-0.66 (m, 10H, -OCH$_2$CH$_2$CH$_2$C*H*$_2$Si-, -SiC*H*$_2$CH$_2$C*H*$_2$Si-), 0.82 (m, 12H, C*H*$_3$CH-), 0.87 (m, 8H, -SiC*H*$_2$CH$_2$S-), 0.94-1.57 (m, 27H, -C*H*$_2$-, -C*H*-, tocopherol, -OCH$_2$CH$_2$C*H*$_2$CH$_2$Si-, -SiCH$_2$C*H*$_2$CH$_2$Si-), 1.19 (s, 3H, -CH$_2$C(C*H*$_3$)OC$_{Ar}$-), 1.75 (m, 4H, -C*H*$_2$C(CH$_3$)OC$_{Ar}$-, -OCH$_2$C*H*$_2$CH$_2$CH$_2$Si-), 2.04 (s, 3H, C*H*$_3$C$_{Ar}$-), 2.08 (s, 3H, C*H*$_3$C$_{Ar}$-), 2.12 (s, 3H, C*H*$_3$C$_{Ar}$-), 2.21 (m, 24H, -N(C*H*$_3$)$_2$), 2.41-2.68 (m, 26H, -C$_{Ar}$C*H*$_2$CH$_2$-, -SiCH$_2$C*H*$_2$S-, -SC*H*$_2$CH$_2$N-, -SCH$_2$C*H*$_2$N-), 3.59 (t, 2H, -OC*H*$_2$CH$_2$CH$_2$CH$_2$Si-). **^{13}C-NMR** ($CDCl_3$): δ (ppm) -5.58 (-Si(CH$_3$)-), -5.43 (-Si(CH$_3$)-), 11.6, 11.9, 12.5 (CH$_3$C$_{Ar}$-), 14.4-14.8 (-SiCH$_2$-), 19.4, 19.6, 20.5, 20.7, 22.4, 22.5, 23.7, 24.5, 24.8, 28.1, 30.9, 32.2, 32.4, 37.2, 37.5, 39.1, 40.1 (-CH$_3$-, -CH$_2$-, -CH-, tocopherol), 21.2 (-SiCH$_2$CH$_2$CH$_2$Si-), 27.6 (-SiCH$_2$CH$_2$S-), 29.4 (-SCH$_2$CH$_2$N-), 29.7 (-OCH$_2$CH$_2$CH$_2$CH$_2$Si-), 33.5 (-OCH$_2$CH$_2$CH$_2$CH$_2$Si-), 49.4 (-N(CH$_3$)$_2$), 59.2 (-SCH$_2$CH$_2$N-), 72.1 (-OCH$_2$CH$_2$CH$_2$CH$_2$Si-), 74.3 (-CH$_2$(CH$_3$)COC$_{Ar}$-), 117.3 (-*C$_{Ar}$*CH$_2$CH$_2$-), 122.6, 125.3, 127.2 (CH$_3$*C$_{Ar}$*-), 147.6 (-*C$_{Ar}$*OC-), 148.3 (-*C$_{Ar}$*OCH$_2$-). **Mass spectroscopy**: $[M+H]^+$ = 1227.8761 Da (calcd. = 1227.8770 Da). **Elemental analysis** $C_{66}H_{134}N_4O_2S_4Si_3$ (1228.32 g/mol): calcd. = C, 64.54; H, 11.00; N, 4.56; O, 2.61; S, 10.44; Si, 6.86. Found = C, 63.90; H, 11.23; N, 4.16; S, 10.37.

Synthesis of $EG_3(SNMe_2)_8$ (15).

Following a similar procedure described for **13**, dendron **15** was obtained as yellow oil (83%) using $EG_3(SNMe_2{\cdot}HCl)_8$ (**12**) (1.9 g; 0.80 mmol), Na_2CO_3 (0.988 g; 9.32 mmol).

^{1}**H-NMR** (CDCl$_3$): δ (ppm) -0.05 (s, 9H, -Si(CH$_3$)-), 0.02 (s, 12H, -Si(CH$_3$)-), 0.51-0.73 (m, 26H, -OCH$_2$CH$_2$CH$_2$CH$_2$Si-, -SiCH$_2$CH$_2$CH$_2$Si-), 0.85 (m, 12H, CH$_3$CH-), 0.89 (m, 16H, -SiCH$_2$CH$_2$S-), 0.95-1.61 (m, 35H, -CH$_2$-, -CH-, tocoferol, -OCH$_2$CH$_2$CH$_2$CH$_2$Si-, -SiCH$_2$CH$_2$CH$_2$Si-), 1.18 (s, 3H, -CH$_2$C(CH$_3$)OC$_{Ar}$-), 1.75 (m, 4H, -CH$_2$C(CH$_3$)OC$_{Ar}$-, -OCH$_2$CH$_2$CH$_2$CH$_2$Si-), 2.06 (s, 3H, CH$_3$C$_{Ar}$-), 2.08 (s, 3H, CH$_3$C$_{Ar}$-), 2.13 (s, 3H, CH$_3$C$_{Ar}$-), 2.21 (m, 48H, -N(CH$_3$)$_2$), 2.40-2.67 (m, 50H, -C$_{Ar}$CH$_2$CH$_2$-, -SiCH$_2$CH$_2$S-, -SCH$_2$CH$_2$N-, -SCH$_2$CH$_2$N-), 3.64 (t, 2H, -OCH$_2$CH$_2$CH$_2$CH$_2$Si-). ^{13}**C-NMR** (CDCl$_3$): δ (ppm) -5.58 (-Si(CH$_3$)-), -5.41 (-Si(CH$_3$)-), 11.7, 12.3, 12.6 (CH$_3$C$_{Ar}$-), 14.1-14.8 (-SiCH$_2$-), 19.3, 19.5, 20.4, 20.6, 22.6, 22.7, 23.6, 24.6, 24.7, 27.9, 31.1, 32.4, 32,6, 37.2, 37,3, 39.1, 40.3 (-CH$_3$-, -CH$_2$-, -CH-, tocopherol), 21.3 (-SiCH$_2$CH$_2$CH$_2$Si-), 27.3 (-SiCH$_2$CH$_2$S-), 29.5 (-SCH$_2$CH$_2$N-), 29.7 (-OCH$_2$CH$_2$CH$_2$CH$_2$Si-), 33.2 (-OCH$_2$CH$_2$CH$_2$CH$_2$Si-), 49.8 (-N(CH$_3$)$_2$), 59.1 (-SCH$_2$CH$_2$N-), 72.6 (-OCH$_2$CH$_2$CH$_2$CH$_2$Si-), 74.4 (-CH$_2$(CH$_3$)COC$_{Ar}$-), 117.4 (-C$_{Ar}$CH$_2$CH$_2$-), 122.7, 125.5, 127.6 (CH$_3$C$_{Ar}$-), 147.7 (-C$_{Ar}$OC-), 148.5 (-C$_{Ar}$OCH$_2$-). **Mass spectroscopy**: [M+H]$^+$ = 2096.4083 Da (calcd. = 2096.4052 Da). **Elemental analysis** $C_{106}H_{226}N_8O_2S_8Si_7$ (2098.10 g/mol): calcd. = C, 60.68; H, 10.86; N, 5.34; O, 1.53; S, 12.22; Si, 9.37. Found = C, 60.59; H, 10.76; N, 5.04; S, 11.99.

Synthesis of EG$_2$(NMe$_3$I)$_4$ (17).

Following the procedure described for **4** or **16**, dendron **17** was obtained as yellow solid (96%) using EG$_2$(NMe$_2$)$_4$ (**14**) (0.50 g, 0.41 mmol), MeI (0.15 mL, 2.41 mmol).

^{1}**H-NMR** (D$_2$O): δ (ppm) 0.08 (s, 3H, -Si(CH$_3$)-), 0.15 (s, 6H, -Si(CH$_3$)-), 0.71-0.83 (m, 10H, -OCH$_2$CH$_2$CH$_2$CH$_2$Si-, -SiCH$_2$CH$_2$CH$_2$Si-), 0.91 (m, 12H, CH$_3$CH-), 1.02 (m, 8H, -SiCH$_2$CH$_2$S-), 1.05-1.73 (m, 27H, -CH$_2$-, -CH-, tocopherol, -OCH$_2$CH$_2$CH$_2$CH$_2$Si-, -SiCH$_2$CH$_2$CH$_2$Si-), 1.23 (s, 3H, -CH$_2$C(CH$_3$)OC$_{Ar}$-), 1.86 (m, 4H, -CH$_2$C(CH$_3$)OC$_{Ar}$-, -OCH$_2$CH$_2$CH$_2$CH$_2$Si-), 2.11 (m, 3H, CH$_3$C$_{Ar}$-), 2.17 (m, 3H, CH$_3$C$_{Ar}$-), 2,19 (m, 3H, CH$_3$C$_{Ar}$-), 2.64 (t, 2H, -C$_{Ar}$CH$_2$CH$_2$-), 2.79 (m, 8H, -SiCH$_2$CH$_2$S-), 2.98 (m, 8H, -SCH$_2$CH$_2$N-), 3.25 (m, 36H, -N(CH$_3$)$_3$), 3.70 (m, 10H, -OCH$_2$CH$_2$CH$_2$CH$_2$Si-, -SCH$_2$CH$_2$N-). ^{13}**C-NMR** (D$_2$O): δ (ppm) -4,38 (-Si(CH$_3$)-), 12.4, 12.6, 13.5 (CH$_3$C$_{Ar}$-), 14.6-15.5 (-SiCH$_2$-), 20.4, 21.6, 21.8, 22.0, 23.3, 23.4, 23.5, 24.5, 25.3, 28.9, 32.5, 34.0, 34.3, 37.4, 38.5, 38.6, 38.7, 40.8 (-CH$_3$-, -CH$_2$-, -CH-, tocopherol), 22.5 (-SiCH$_2$CH$_2$CH$_2$Si-), 26.0 (-SiCH$_2$CH$_2$S-), 29.2 (-SCH$_2$CH$_2$N-), 31.6 (-OCH$_2$CH$_2$CH$_2$CH$_2$Si-), 35,3 (-OCH$_2$CH$_2$CH$_2$CH$_2$Si-), 54.2 (-N(CH$_3$)$_3$), 67.6 (-SCH$_2$CH$_2$N-), 73.4 (-OCH$_2$CH$_2$CH$_2$CH$_2$Si-), 75.7 (-CH$_2$(CH$_3$)COC$_{Ar}$-), 118.3 (-C$_{Ar}$CH$_2$CH$_2$-), 123.6, 126.7, 128.0 (CH$_3$C$_{Ar}$-), 148.7 (-C$_{Ar}$OC-), 149.6 (-C$_{Ar}$OCH$_2$-). **Mass spectroscopy**: [tocoferol+H]$^+$ = 431.3903 Da (calcd. = 431.3884

Da); [M-I-tocoferol]$^+$ = 1255.3022 Da (calcd. = 1255.3065 Da). **Elemental analysis** $C_{70}H_{146}I_4N_4O_2S_4Si_3$ (1796.08 g/mol): calcd. = C, 46.81; H, 8.19; I, 28.26; N, 3.12; O, 1.78; S, 7.14; Si, 4.69. Found= C, 46.45; H, 7.79; N, 2.62; S, 6.80.

Synthesis of EG$_3$(NMe$_3$I)$_8$ (18).

Following the procedure described for **4** or **16**, dendron **18** was obtained as yellow solid (96%) using EG$_3$(NMe$_2$)$_8$ (**15**) (0.50 g, 0.24 mmol), MeI (0.20 mL, 3.21 mmol).

^{1}H-NMR (D_2O): δ (ppm) 0.08 (s, 9H, -Si(C*H*$_3$)-), 0.17 (s, 12H, -Si(C*H*$_3$)-), 0.68-0.85 (m, 26H, -OCH$_2$CH$_2$CH$_2$C*H*$_2$Si-, -SiC*H*$_2$CH$_2$C*H*$_2$Si-), 0.91 (m, 12H, C*H*$_3$CH-), 1.04 (m, 16H, -SiC*H*$_2$CH$_2$S-), 1.08-1.79 (m, 35H, -C*H*$_2$-, -C*H*-, tocopherol, -OCH$_2$CH$_2$C*H*$_2$CH$_2$Si-, -SiCH$_2$C*H*$_2$CH$_2$Si-), 1.27 (s, 3H, -CH$_2$C(C*H*$_3$)OC$_{Ar}$-), 1.84 (m, 4H, -C*H*$_2$C(CH$_3$)OC$_{Ar}$-, -OCH$_2$C*H*$_2$CH$_2$CH$_2$Si-), 2.10 (m, 3H, C*H*$_3$C$_{Ar}$-), 2.15 (m, 3H, C*H*$_3$C$_{Ar}$-), 2.18 (m, 3H, C*H*$_3$C$_{Ar}$-), 2.68 (t, 2H, -C$_{Ar}$C*H*$_2$CH$_2$-), 2.81 (m, 16H, -SiCH$_2$C*H*$_2$S-), 3.03 (m, 16H, -SC*H*$_2$CH$_2$N-), 3.18 (m, 72H, -N(C*H*$_3$)$_3$), 3.67 (m, 18H, -OC*H*$_2$CH$_2$CH$_2$CH$_2$Si-, -SCH$_2$C*H*$_2$N-). **^{13}C-NMR** (D_2O): δ (ppm) -4.36 (-Si(CH$_3$)-), 12.3, 12.7, 13.6 (CH$_3$C$_{Ar}$-), 14.9-15.6 (-SiCH$_2$-), 20.3, 21.6, 21.7, 22.0, 23.1, 23.3, 23.4, 24.7, 25.2, 29.1, 32.3, 33.8, 34.2, 37.6, 38.5, 38.7, 38.9, 41.0 (-CH$_3$-, -CH$_2$-, -CH-, tocopherol), 22.6 (-SiCH$_2$CH$_2$CH$_2$Si-), 26.3 (-SiCH$_2$CH$_2$S-), 29.4 (-SCH$_2$CH$_2$N-), 31.3 (-OCH$_2$CH$_2$CH$_2$CH$_2$Si-), 35.2 (-OCH$_2$CH$_2$CH$_2$CH$_2$Si-), 54.3 (-N(CH$_3$)$_3$), 67.4 (-SCH$_2$CH$_2$N-), 73.7 (-OCH$_2$CH$_2$CH$_2$CH$_2$Si-), 75.9 (-CH$_2$(CH$_3$)COC$_{Ar}$-), 118.6 (-*C$_{Ar}$*CH$_2$CH$_2$-), 123.3, 126.8, 127.8 (CH$_3$*C$_{Ar}$*-), 148.8 (-*C$_{Ar}$*OC-), 150.0 (-*C$_{Ar}$*OCH$_2$-). **Mass spectroscopy**: [tocopherol+H]$^+$ = 431.3891 Da (calcd. = 431.3884 Da); [M-I-tocopherol]$^+$ = 2691.5411 Da (calcd. = 2691.5465 Da). **Elemental analysis** $C_{114}H_{250}I_8N_8O_2S_8Si_7$ (3233.62 g/mol): calcd. = C, 42.34; H, 7.79; I, 31.40; N, 3.47; O, 0.99; S, 7.93; Si, 6.08. Found = C, 42.60; H, 7,93; N, 3.18; S, 7.62.

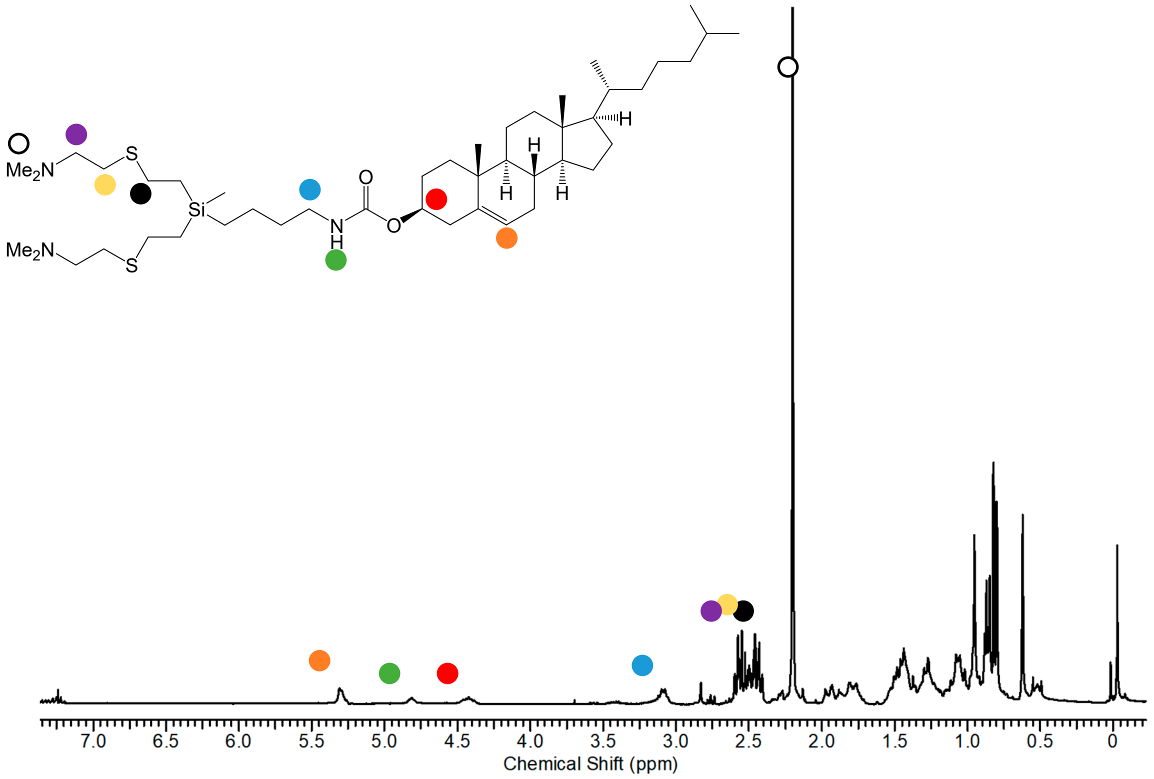

Figure S1. ^{1}H-NMR spectrum of dendron ChG$_1$(SNMe$_2$)$_2$ (**1**) in CDCl$_3$

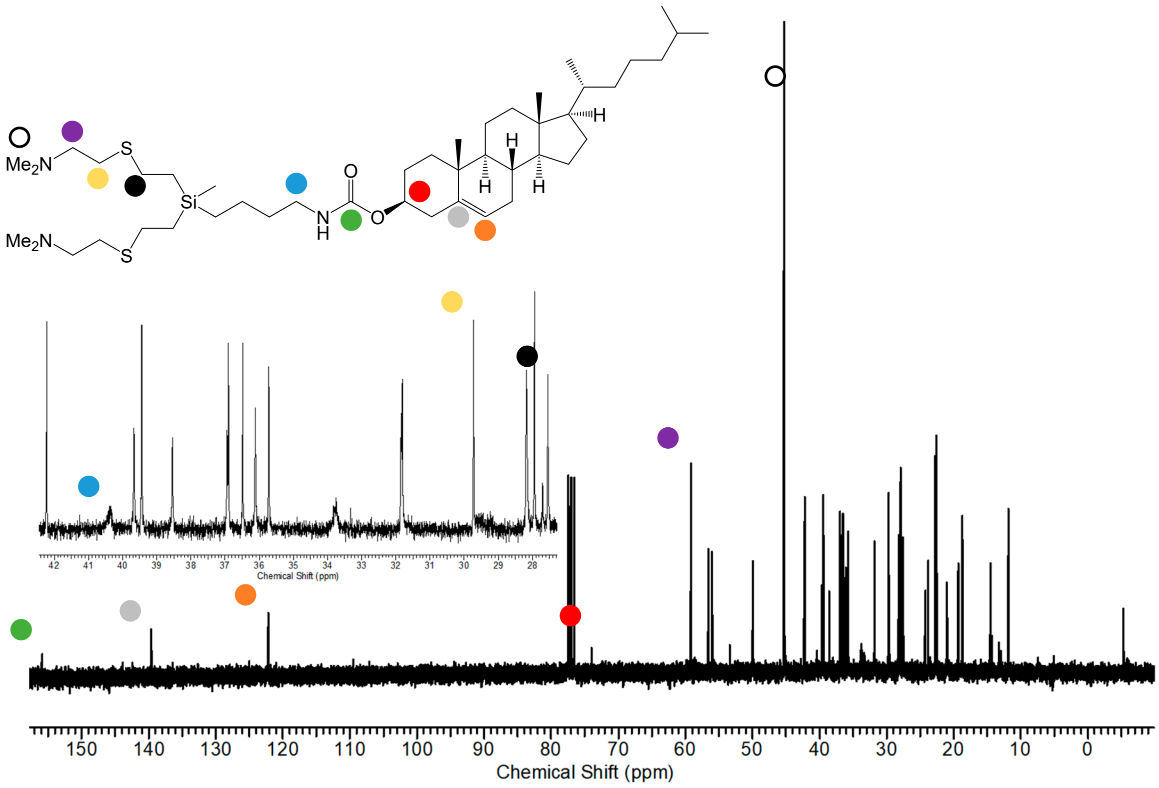

Figure S2. ^{13}C-NMR spectrum of dendron ChG$_1$(SNMe$_2$)$_2$ (**1**) in CDCl$_3$

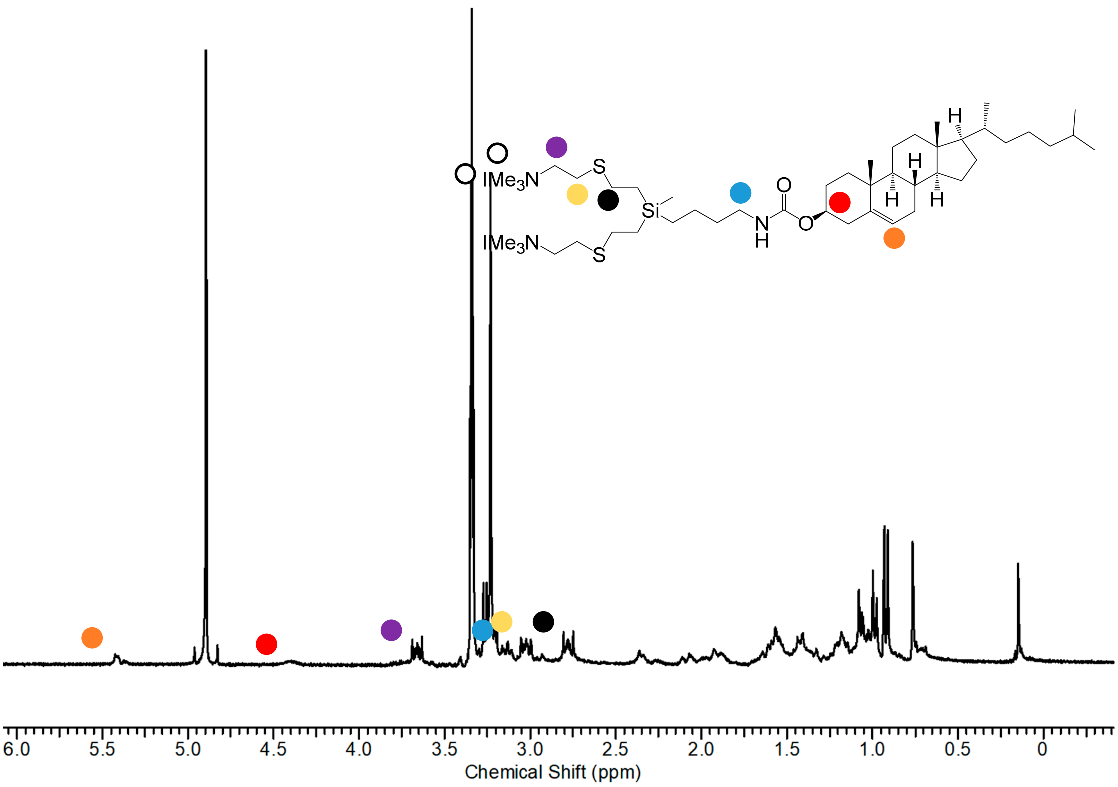

Figure S3. ^{1}H-NMR spectrum of dendron ChG$_1$(SNMe$_3$I)$_2$ (**4**) in CD$_3$OD

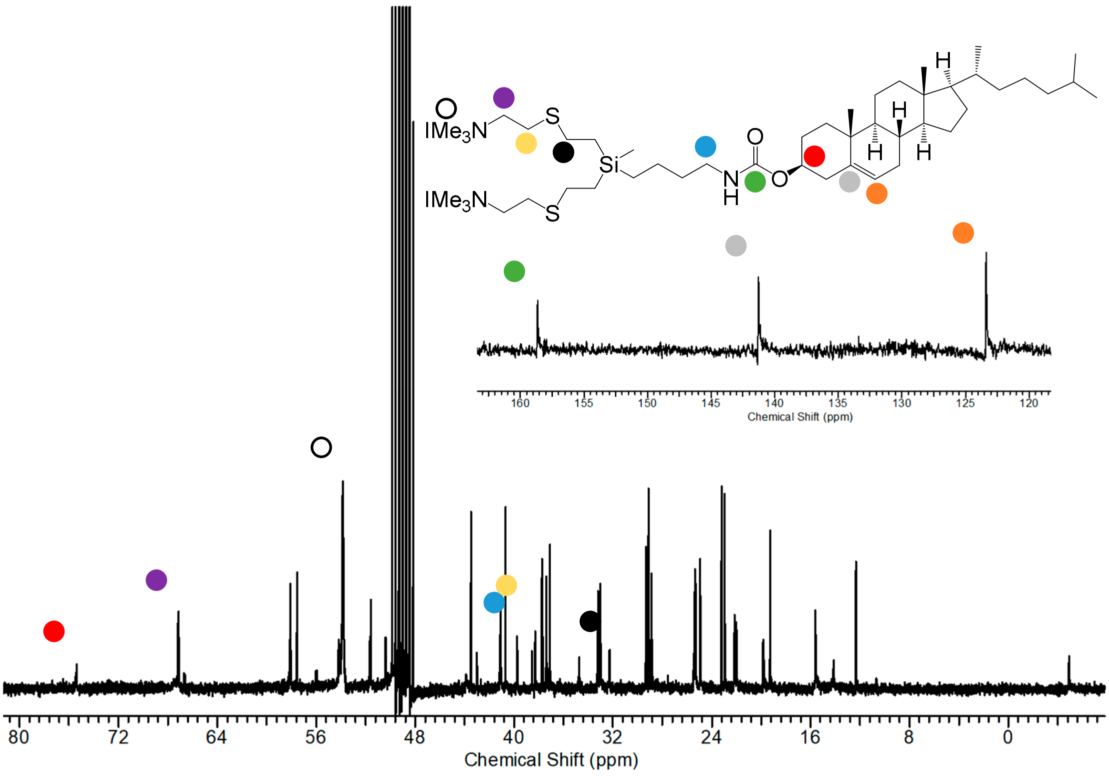

Figure S4. ^{13}C-NMR spectrum of dendron ChG$_1$(SNMe$_3$I)$_2$ (**4**) in CD$_3$OD

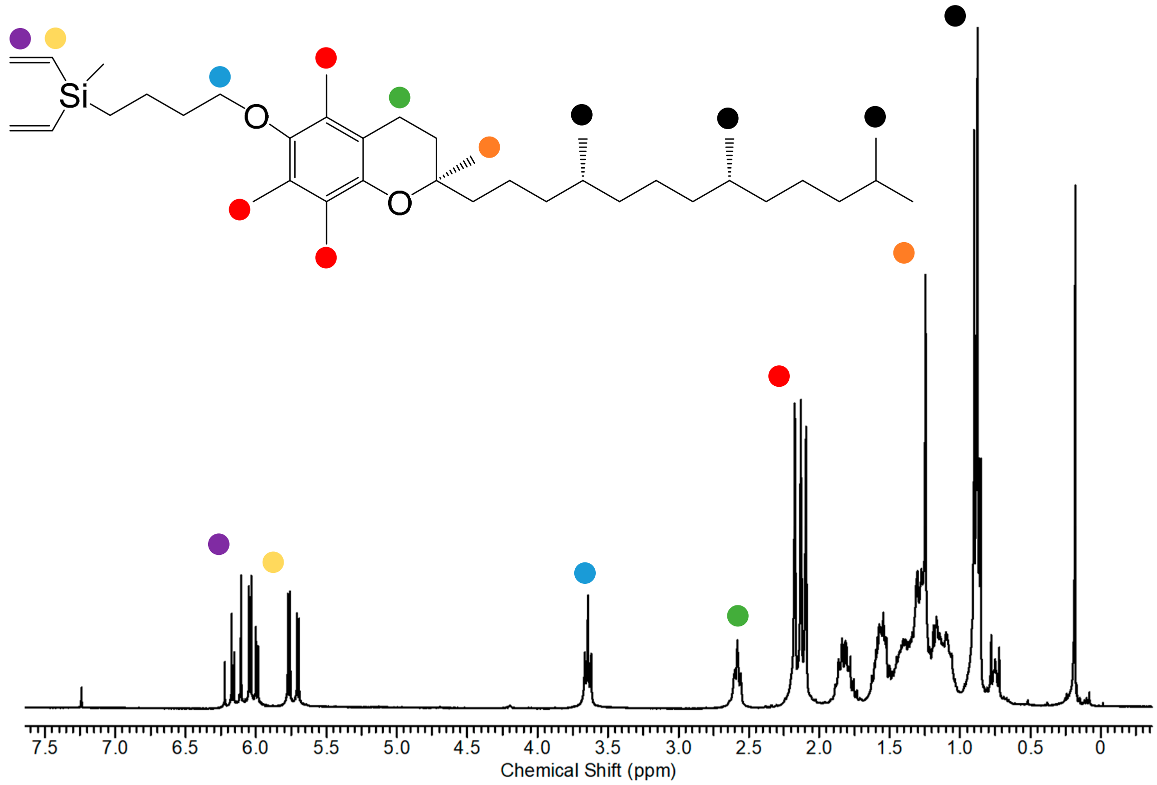

Figure S5. ^{1}H-NMR spectrum of dendron $EG_1(V)_2$ (**7**) in $CDCl_3$

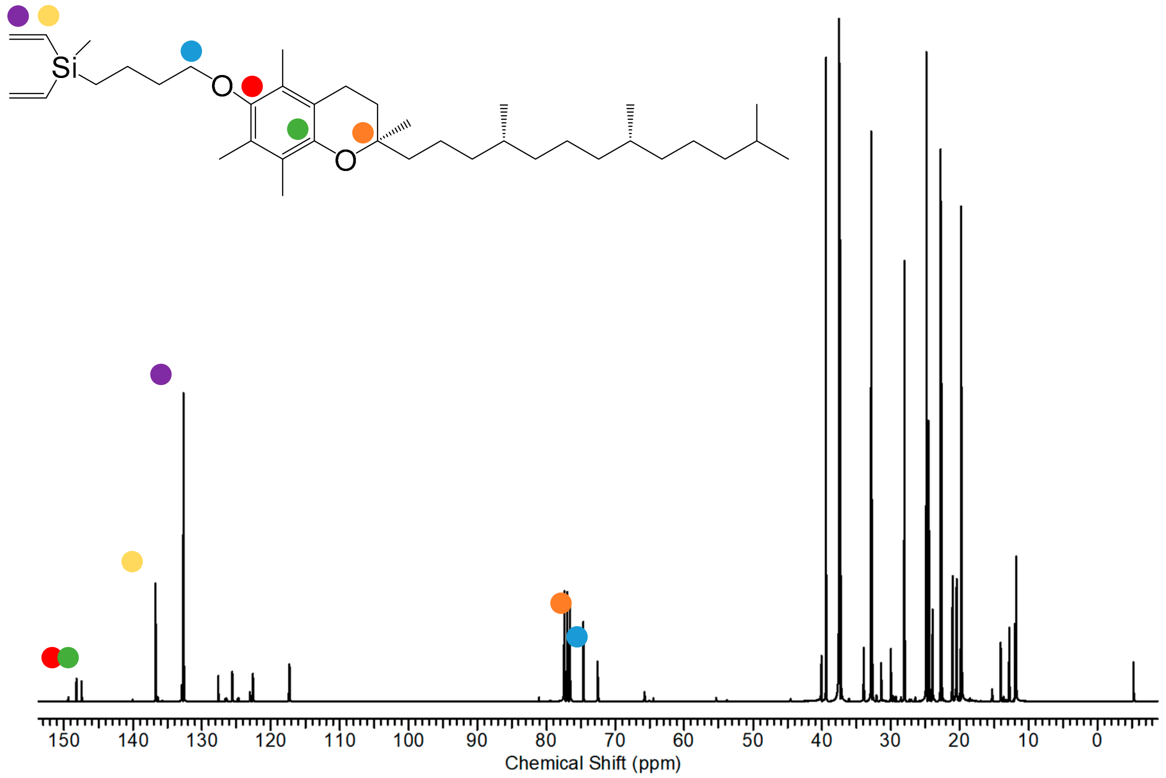

Figure S6. ^{13}C-NMR spectrum of dendron $EG_1(V)_2$ (**7**) in $CDCl_3$

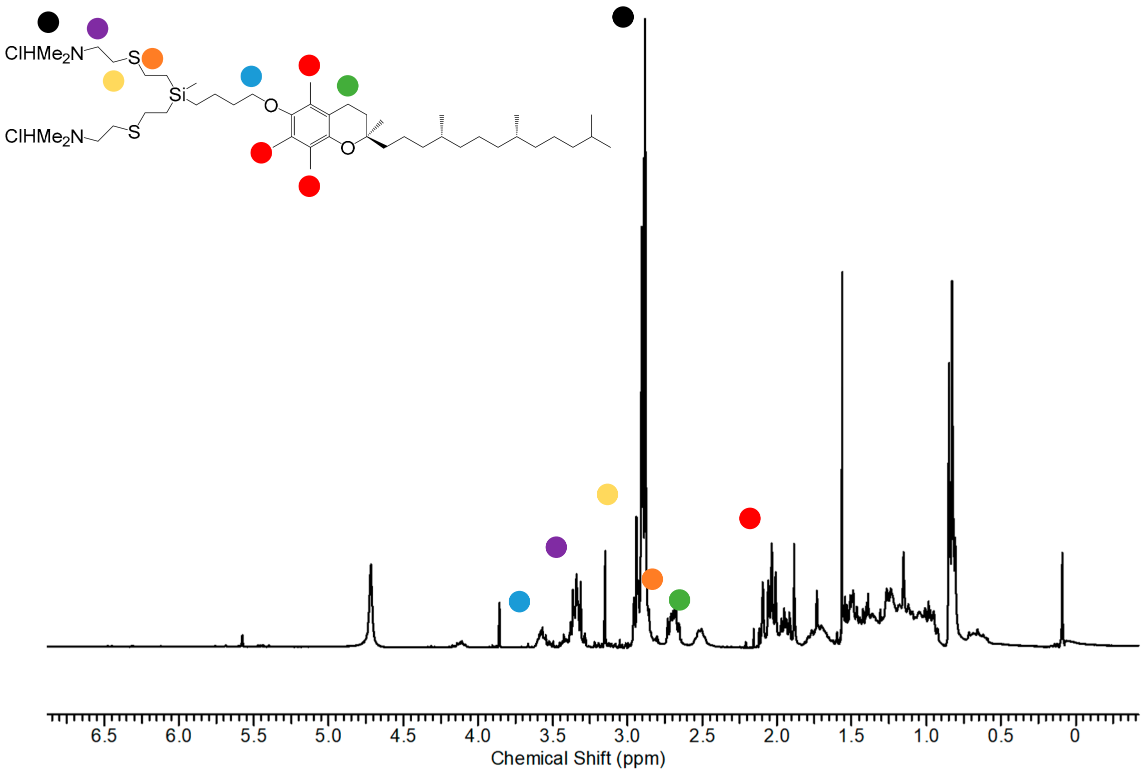

Figure S7. ^{1}H-NMR spectrum of dendron $EG_1(SNMe_2{\cdot}HCl)_2$ (**10**) in CD_3OD

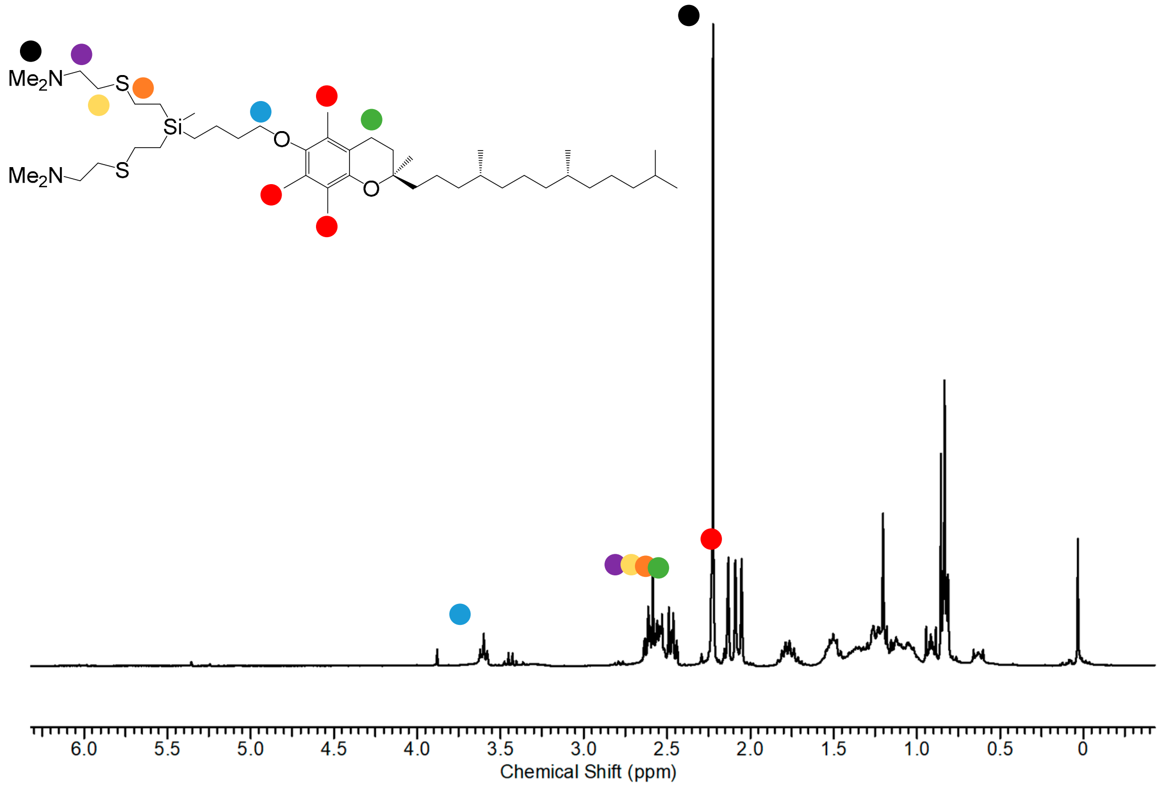

Figure S8. ^{1}H-NMR spectrum of dendron $EG_1(SNMe_2)_2$ (**13**) in $CDCl_3$

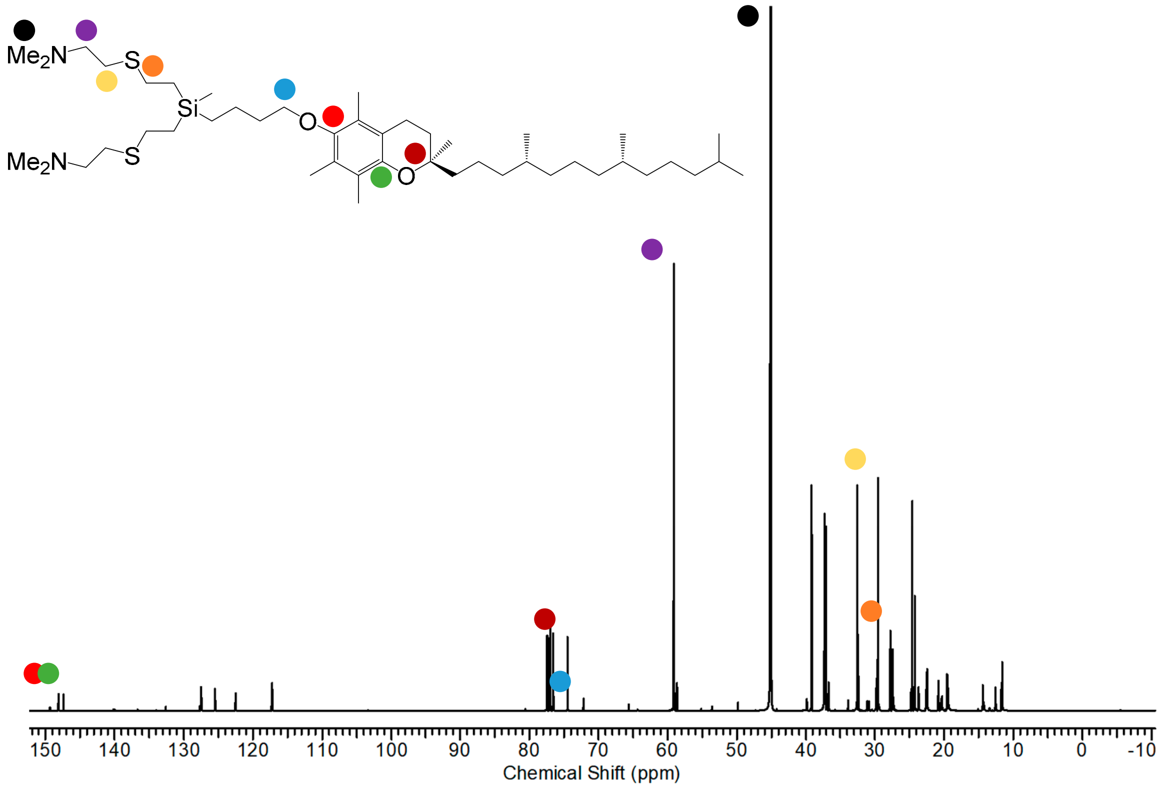

Figure S9. ^{13}C-NMR spectrum of dendron EG$_1$(SNMe$_2$)$_2$ (**13**) in CDCl$_3$

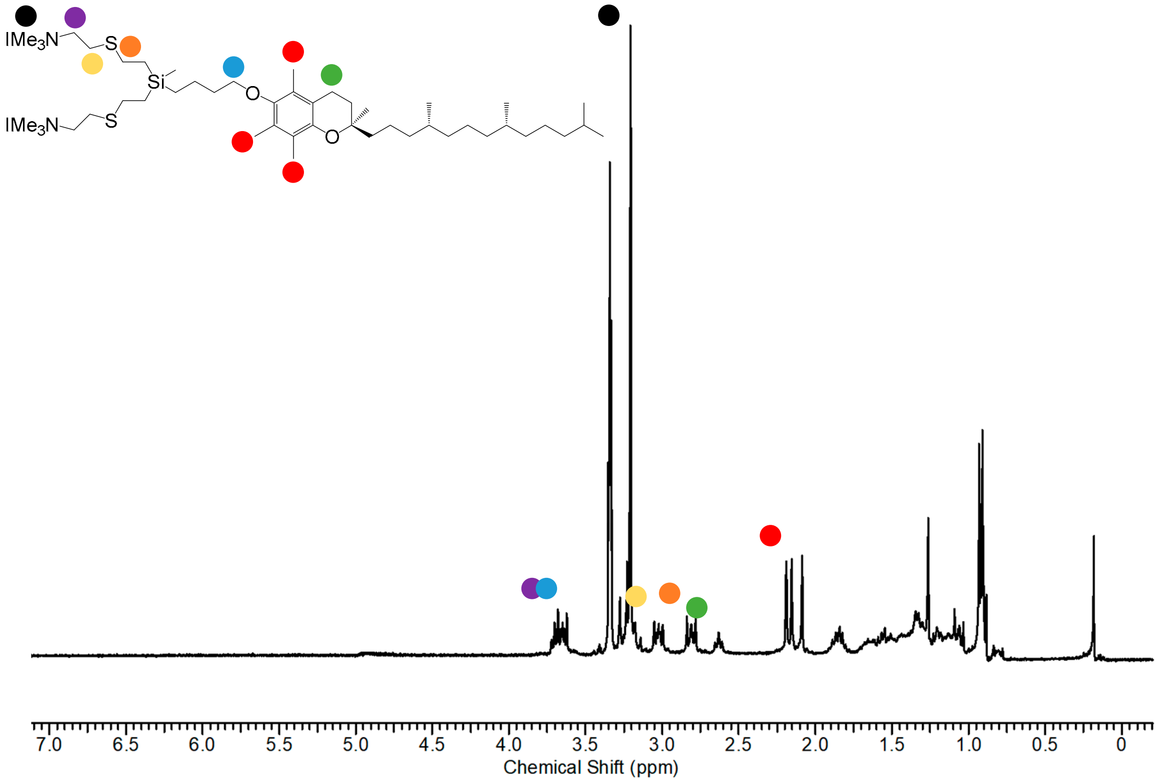

Figure S10. ^{1}H-NMR spectrum of dendron EG$_1$(SNMe$_3$I)$_2$ (**16**) in CD$_3$OD

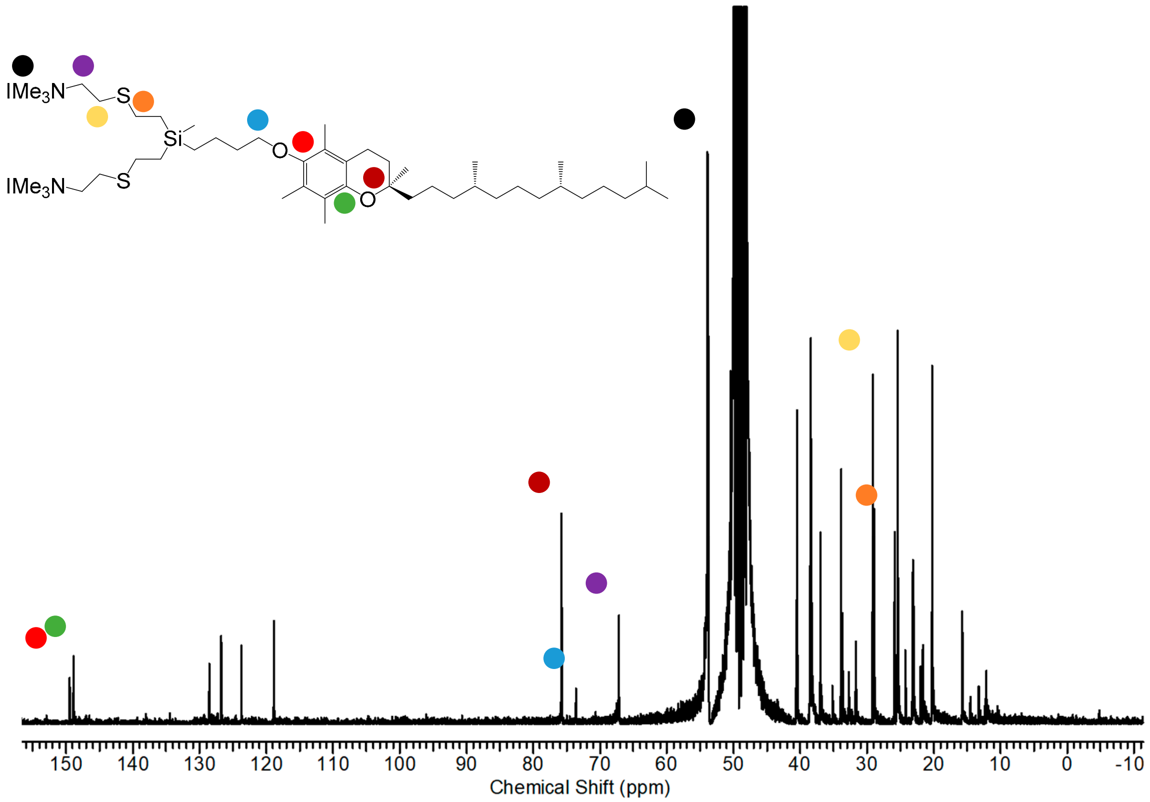

Figure S11. ^{13}C-NMR spectrum of dendron $EG_1(SNMe_3I)_2$ (**16**) in CD_3OD

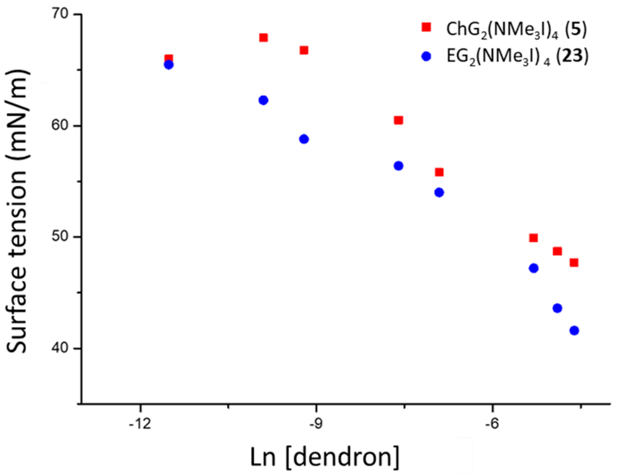

Figure S12. Surface tension measurements of dendrons $ChG_2(SNMe_3I)_4$ (**5**) and $EG_2(SNMe_3I)_4$ (**17**) at increasing concentrations without the presence of salt

Table S1. Surface tension values of 1 mM solution of dendrons **5** and **17** at different concentrations of NaCl and NaI

Salt Concentration (mM)	Surface tension 5 + NaCl (mN/m)	Surface tension 5 + NaI (mN/m)	Surface tension 17 + NaCl (mN/m)	Surface tension 17 + NaI (mN/m)
0	55.8	55.8	54.0	54.0
20	49.0	45.2	47.9	44.8
40	48.1	43.9	44.6	42.8

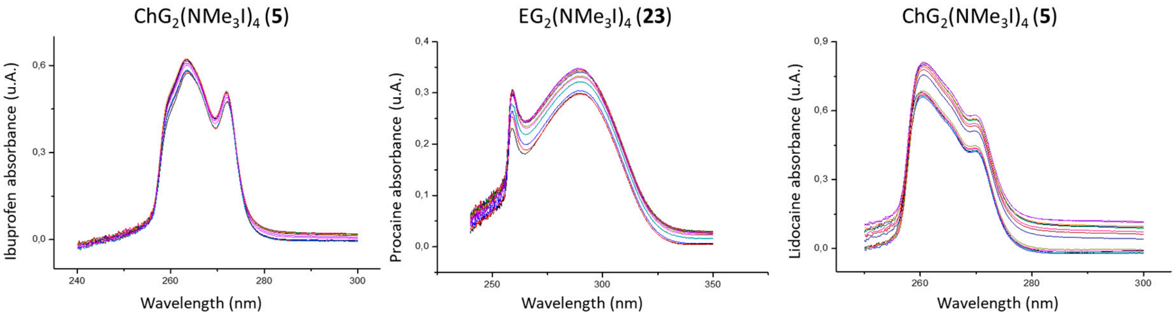

Figure S13. UV-Vis absorption spectra of saline solution (40 mM of NaI) of increasing concentrations of dendrons **5** and **17** in presence of ibuprofen (1.5 mM), procaine (1.5 mM) or lidocaine (10 µM).

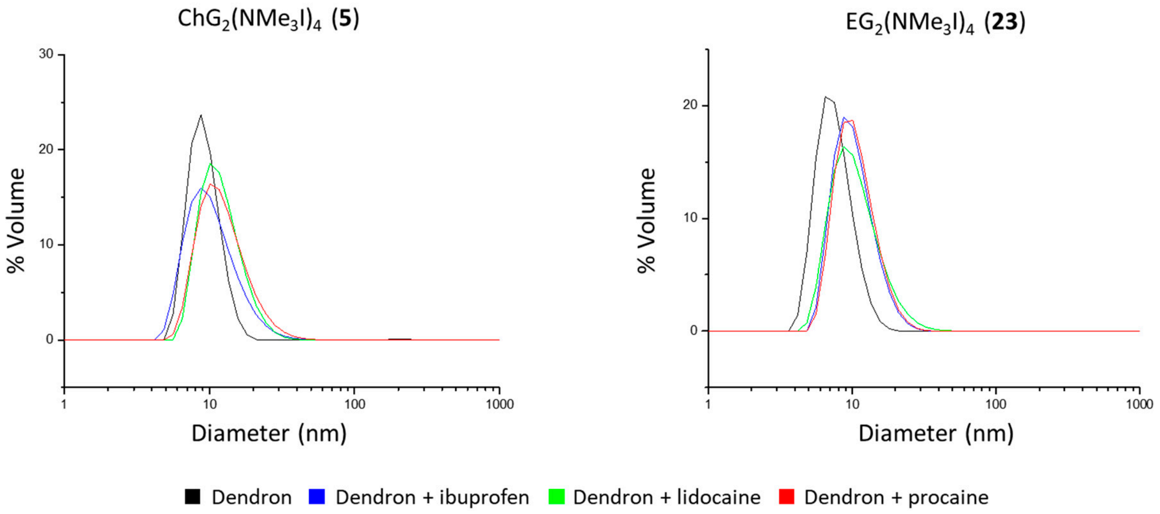

Figure S14. Particle size distribution of amphiphilic dendrons **5** and **17** in absence or presence of ibuprofen (1.5 mM), lidocaine (1.5 mM) or procaine (10 µM) with 40 mM of NaI.

Solubility determination for hydrophobic drugs. For the determination of solubility, excess of drug was placed in contact with dendron 5 or 17 (0.5 – 5.5 mM) in 2 mL of saline aqueous solution (NaI 40mM). The flasks were maintained at 25°C and were stirred for 24 h. After that, the solutions were filtered (0.2 µm CA filter). The samples were analyzed by HPLC and the concentrations of solubilized drug was determined by HPLC (as described above).

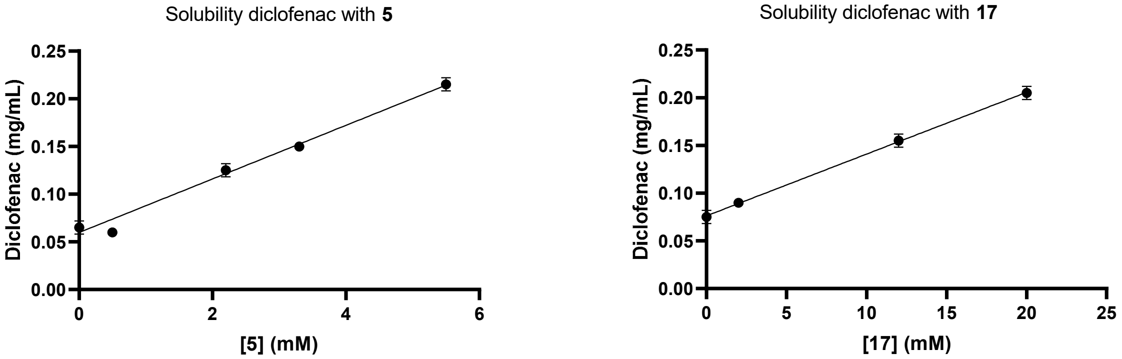

Figure S15. Solubilization of diclofenac by dendron **5** or **17.**

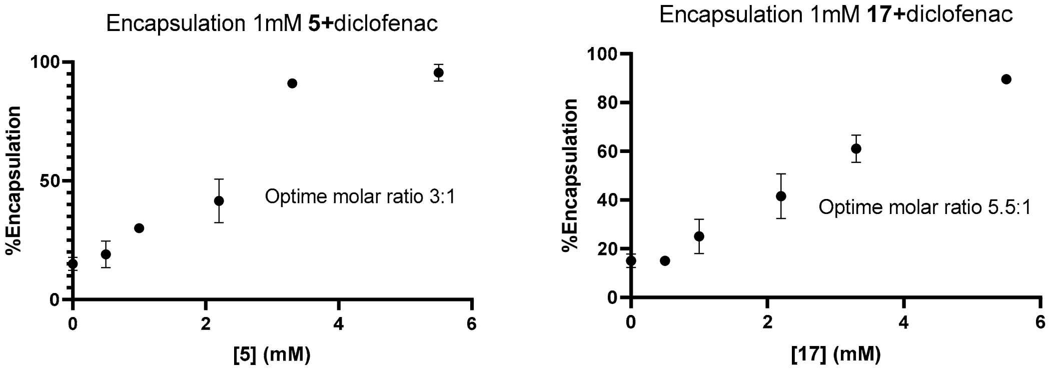

Figure S16. Relation between dendron **5** or **17** and encapsulation percentage at 1 mM diclofenac. Optime dendron:diclofenac molar ratio 3.3:1 (**5**) and 5.5:1 (**17**).

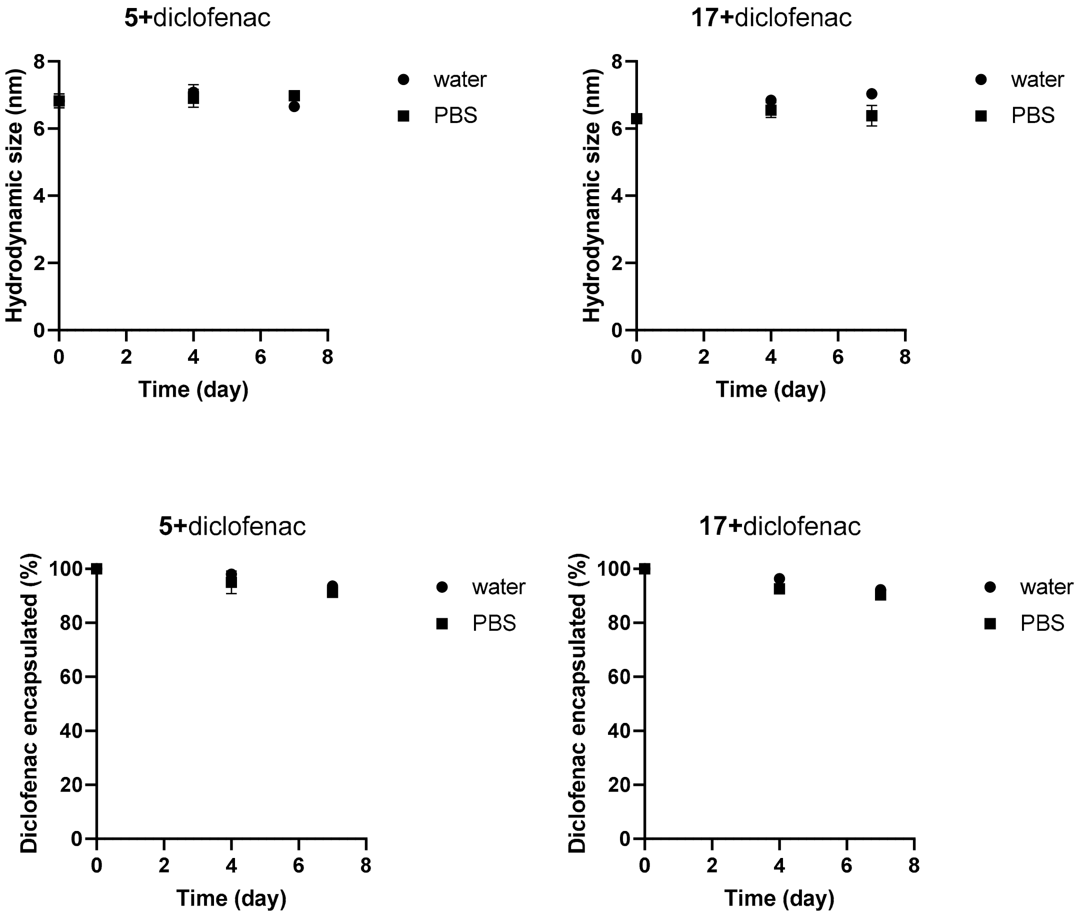

Figure S17. Time-dependent hydrodynamic size and % diclofenac encapsulated change for **5**+diclofenac and **17**+diclofenac micelles dispersed in water or PBS (10 mM).
